# Participation in adherence clubs and on-time drug pickup among HIV-infected adults in Zambia: A matched-pair cluster randomized trial

**DOI:** 10.1371/journal.pmed.1003116

**Published:** 2020-07-01

**Authors:** Monika Roy, Carolyn Bolton-Moore, Izukanji Sikazwe, Mpande Mukumbwa-Mwenechanya, Emilie Efronson, Chanda Mwamba, Paul Somwe, Estella Kalunkumya, Mwansa Lumpa, Anjali Sharma, Jake Pry, Wilbroad Mutale, Peter Ehrenkranz, David V. Glidden, Nancy Padian, Stephanie Topp, Elvin Geng, Charles B. Holmes

**Affiliations:** 1 University of California, San Francisco, San Fancisco, California, United States of America; 2 Centre for Infectious Disease Research in Zambia, Lusaka, Zambia; 3 University of Alabama, Tuscaloosa, Alabama, United States of America; 4 University of California, Davis, Davis, California, United States of America; 5 Bill and Melinda Gates Foundation, Seattle, Washington, United States of America; 6 University of California, Berkeley, Berkeley, California, United States of America; 7 James Cook University, Townsville, Queensland, Australia; 8 Johns Hopkins University, Baltimore, Maryland, United States of America; 9 Center for Global Health Practice and Impact, Georgetown University School of Medicine, Washington, District of Columbia, United States of America; Boston University, UNITED STATES

## Abstract

**Background:**

Current models of HIV service delivery, with frequent facility visits, have led to facility congestion, patient and healthcare provider dissatisfaction, and suboptimal quality of services and retention in care. The Zambian urban adherence club (AC) is a health service innovation designed to improve on-time drug pickup and retention in HIV care through off-hours facility access and pharmacist-led group drug distribution. Similar models of differentiated service delivery (DSD) have shown promise in South Africa, but observational analyses of these models are prone to bias and confounding. We sought to evaluate the effectiveness and implementation of ACs in Zambia using a more rigorous study design.

**Methods and findings:**

Using a matched-pair cluster randomized study design (ClinicalTrials.gov: NCT02776254), 10 clinics were randomized to intervention (5 clinics) or control (5 clinics). At each clinic, between May 19 and October 27, 2016, a systematic random sample was assessed for eligibility (HIV+, age ≥ 14 years, on ART >6 months, not acutely ill, CD4 count not <200 cells/mm^3^) and willingness to participate in an AC. Clinical and antiretroviral drug pickup data were obtained through the existing electronic medical record. AC meeting attendance data were collected at intervention facilities prospectively through October 28, 2017. The primary outcome was time to first late drug pickup (>7 days late). Intervention effect was estimated using unadjusted Kaplan–Meier survival curves and a Cox proportional hazards model to derive an adjusted hazard ratio (aHR). Medication possession ratio (MPR) and implementation outcomes (adoption, acceptability, appropriateness, feasibility, and fidelity) were additionally evaluated as secondary outcomes. Baseline characteristics were similar between 571 intervention and 489 control participants with respect to median age (42 versus 41 years), sex (62% versus 66% female), median time since ART initiation (4.8 versus 5.0 years), median CD4 count at study enrollment (506 versus 533 cells/mm^3^), and baseline retention (53% versus 55% with at least 1 late drug pickup in previous 12 months). The rate of late drug pickup was lower in intervention participants compared to control participants (aHR 0.26, 95% CI 0.15–0.45, *p* < 0.001). Median MPR was 100% in intervention participants compared to 96% in control participants (*p <* 0.001). Although 18% (683/3,734) of AC group meeting visits were missed, on-time drug pickup (within 7 days) still occurred in 51% (350/683) of these missed visits through alternate means (use of buddy pickup or early return to the facility). Qualitative evaluation suggests that the intervention was acceptable to both patients and providers. While patients embraced the convenience and patient-centeredness of the model, preference for traditional adherence counseling and need for greater human resources influenced intervention appropriateness and feasibility from the provider perspective. The main limitations of this study were the small number of clusters, lack of viral load data, and relatively short follow-up period.

**Conclusions:**

ACs were found to be an effective model of service delivery for reducing late ART drug pickup among HIV-infected adults in Zambia. Drug pickup outside of group meetings was relatively common and underscores the need for DSD models to be flexible and patient-centered if they are to be effective.

**Trial registration:**

ClinicalTrials.gov NCT02776254.

## Abstract 2

Monika Roy and colleagues study methods to streamline delivery of antiretrovirals to people with HIV infection in Zambia.

## Author summary

**Why was this study done?:**

- The adherence club intervention was designed to decrease burden on the health system and improve retention in care among treatment-experienced, clinically stable HIV-infected adults by providing off-hours facility access and streamlined (group-based, pharmacist-led) drug delivery.
- To date, published data on adherence clubs have been primarily limited to observational data from a similar intervention in South Africa.
- This study was done to learn about the effectiveness of the intervention and challenges with implementation in other settings in sub-Saharan Africa.

**What did the researchers do and find?:**

- We conducted a matched-pair cluster randomized study to estimate the effect of participating in adherence clubs on on-time drug pickup in Zambia.
- We additionally used a mixed-methods approach to evaluate the implementation of the intervention.
- We found that participation in the adherence club led to a reduction in the occurrence and rate of experiencing a late drug pickup. However, on-time drug pickups outside of adherence club meetings were relatively common.
- Both patients and providers found the intervention to be acceptable, but while patients embraced the patient-centered aspects of adherence clubs, some providers questioned the appropriateness and feasibility of the model.

**What do these findings mean?:**

- The urban adherence club intervention decreased late drug pickup among treatment-experienced adults living with HIV in Zambia.
- However, patients commonly missed their club meeting, seeking alternative means to pick up their medication on time.
- While club participation was associated with greater retention in care, flexibility in drug pickup and patient-centeredness were likely important factors.

## Introduction

Traditional facility-based models of HIV care in sub-Saharan Africa are marked by frequent clinic visits requiring considerable time investment, travel distance, and cost to the patient [1–4]. This has led to suboptimal downstream consequences for both patients and health systems, including poor long-term retention in care (less than 70% at 2 years after ART initiation) [5] and exacerbation of existing infrastructure and human resource shortages, leading to clinic wait times often exceeding 4 hours [6–8]. The concept of differentiated service delivery (DSD) was developed in response to these challenges and to provide greater patient-centered care among HIV-infected individuals accessing lifelong antiretroviral therapy (ART) [9,10]. DSD models, which seek to tailor the intensity, frequency, and location of HIV care to patient characteristics and needs, are currently being tested and scaled up throughout sub-Saharan Africa [9,10].

The ART adherence club (AC) is one of several DSD models originally developed by Médecins Sans Frontières in South Africa with the aim of addressing HIV care delivery challenges through off-hours patient access to the facility, group drug pickup and counseling, and pharmacy and clinical visit spacing. Studies of AC outcomes in South Africa have reported high rates of retention in care (>90% at 24 months) and substantial reductions in loss to follow-up and virological rebound [11–13]. Although promising, the applicability of these findings or the models of care outside of South African study settings is unclear, as data are based primarily on observational analyses in South Africa [14], where the implementation context can be quite different than elsewhere in the region. Assessing the effectiveness of the AC model has become increasingly critical as programs and policymakers throughout sub-Saharan Africa are making decisions regarding specific DSD models of care they wish to adopt at scale. Zambia adapted South Africa’s AC model from a nurse-led to a pharmacist-led intervention with distinct criteria for drug pickup by a buddy and club removal/referral back to facility-based care to address the needs of patients in busy urban settings. There are to our knowledge no published data outside of South Africa evaluating the effectiveness of ACs, and much remains to be understood about the implementability of the model in the region.

To address this gap, we conducted a matched-pair cluster randomized study to evaluate the implementation and effectiveness of ACs in Zambia, a country with a substantial burden of HIV disease (estimated adult prevalence 12.4% in 2017) and a large national HIV prevention, care, and treatment program, with over 750,000 patients estimated to be on treatment [15]. In addition to evaluating retention, using time to first late drug pickup and medication possession ratio (MPR), we employed mixed methods to evaluate key implementation outcomes including patient and healthcare worker acceptability, appropriateness, feasibility, and fidelity.

## Methods

### Description of the intervention

An AC is a group of approximately 30 HIV-positive individuals on ART who meet every 2 months in the first 6 months and every 3 months thereafter, during off-hours at the facility (evenings or weekends) to receive medication refills, symptom screening, and group psychosocial support. Unlike nurse-led South African ACs, the Zambian ACs in this study were led by pharmacy technologists, who were responsible for prepackaging ART medication prior to the meeting and dispensing the ART medication to the members. Groups were supported by 2 community lay health workers who were responsible for conducting symptom screening and leading a group counseling session. In contrast to South African ACs, in which blood samples are taken for analysis at the meeting, and follow-up with a medical officer may occur only once a year, Zambian AC members continued to visit the facility every 6 months for both clinical follow-up and laboratory monitoring. Patients who were unable to attend the meeting in person were allowed to send a buddy for drug pickup on their behalf. In contrast to South African ACs, no restrictions were placed on the frequency of use of the buddy system, and lack of meeting attendance was not considered a criterion for removal from the club and referral back to facility-based care. Patients were up-referred from the AC to the facility in the case of acute illness or positive symptom screening and were transferred out of the AC if they became pregnant or because of patient or clinician preference. The pharmacy technologist and lay healthcare workers were employed by the study; however, the remainder of the model components were supported by existing clinic staff to facilitate integration into routine clinical activities.

### Description of the control

At the time of this study, patients receiving standard of care were assigned return visits to the facility every 1 to 3 months. In addition to seeing the clinician, the patient had separate encounters with the pharmacist and adherence counselor on the day of their visit. Patients may wait, on average, an additional 1.5 hours for each of these encounters [16].

### Setting

The Centre for Infectious Disease Research in Zambia (CIDRZ) is an independent, Zambian non-governmental organization that supports a wide range of national health programs, provides public health and research training, and conducts research. CIDRZ, in collaboration with the Ministry of Health, supports HIV prevention, care, and treatment services in public sector clinics across 4 of 10 provinces in Zambia, using PEPFAR/CDC funding.

### Study design and site selection

All CIDRZ-supported urban clinics in 3 provinces (Lusaka, Southern, and Eastern) that were not part of a large ongoing randomized trial (HPTN 071 [11865]), were considered for inclusion in this study. Clinics were pair-matched on several criteria (clinic province, population size, and baseline patient retention in care) to maximize efficiency and power with a small sample size of clusters. Sample size was derived using formulae for matched-pair cluster randomized trials as specified by Hayes and Moulton [17] and programmed in R 3.2.2 [18]:

$$c={2+(z_{\alpha /2}+z_{\beta })}^{2}\frac{\pi_{0}(1-\pi_{0})/m+\pi_{1}(1-\pi_{1})/m+k_{m}^{2}(\pi_{0}^{2}+\pi_{1}^{2})}{{\left(\pi_{0}-\pi_{1}\right)}^{2}}$$

*k*_*m*_ refers to the matched-pair coefficient of variation. Matched-pair coefficient, number of clinics, and number of patients per clinic were varied to estimate their effect on power (S1 Table). Existing program data suggest that 65% of patients are >7 days late for a pharmacy refill visit at least once during their first year after starting ART, and that 95% of clinics fall within 15% of this estimate (65% ± 15% = 50%–80%). Thus, we assumed a conservative matched-pair coefficient of variation of 0.10. We also assumed a 50% relative reduction in missed pharmacy visits due to AC participation. Under these assumptions, our selection of 5 pairs of clinics and 120 patients per clinic yields a power of 96%.

Five matched pairs of clinics were purposively selected, and then clinics were randomized within each pair (using Stata 14.0) by study investigators to either receive the intervention or serve as a control facility. A systematic random sample of patients (every *n*th patient based on clinic population size) meeting eligibility criteria at intervention and control facilities were assessed for willingness to participate in an AC and, if willing, underwent individual informed consent. The creation of a counterfactual (i.e., comparison of patients who agreed to be in the model at both intervention and control sites) was employed to minimize selection bias. Target enrollment at each clinic was 120 individuals. Individuals at intervention clinics were allocated to 1 of 4 AC groups (30 individuals per group) at that clinic based on patient preference for meeting time. Individuals at control clinics were informed, at the time of informed consent, that the intervention was not currently being offered at their site but may be offered in the future. Qualitative data collection consisted of focus groups and in-depth interviews with patients, healthcare workers, and study staff at all intervention sites.

### Patient recruitment and eligibility

Within both intervention and control facilities, a systematic random sample of eligible patients (HIV positive, age ≥ 14 years, on ART >6 months, not acutely ill, and CD4 count not <200 cells/mm^3^) were assessed for willingness to participate in an AC. Every sixth patient (clinic population greater than 4,000) or every patient (clinic population less than 4,000) who presented for a routine HIV clinical visit was evaluated for eligibility by the HIV clinician. If eligible, the patient was approached by study staff to assess for willingness to join an AC, using an infographic to describe the intervention. Patients who expressed interest subsequently underwent individual written informed consent. Participants were recruited between May 19 and October 27, 2016, and followed until October 28, 2017 (minimum 12 months of follow-up).

Qualitative data were obtained at intervention sites using in-depth interviews and focus groups that were conducted at the midline (January to April 2017) and endline (August to November 2017) of study implementation. A total of 15 focus groups (3 per site) and 16 interviews were conducted at midline, and 20 focus groups (4 per site) and 13 interviews were conducted at endline. Study patients and healthcare workers were invited to participate in focus group discussions via open invitation (during AC meetings or drug pickup for patients and during staff announcements for study staff). For in-depth interviews, facility and study staff were invited to participate either in person or by phone utilizing existing contact information held at the facilities. In-depth interviews were conducted with a purposive sample of ART clinic in-charges, pharmacy technologists, study community liaison officers, study lay healthcare workers, and assistant study coordinators.

### Measurements

We extracted sociodemographic, laboratory, clinical, and drug pickup data for all participants from Smartcare, an existing national electronic medical record used in routine clinical care. Illness at care enrollment was defined as the presence of either WHO stage III or IV disease at HIV care enrollment or an initial CD4 count < 200 cells/mm^3^. For all participants, drug pickup date and next assigned drug pickup date in Smartcare were utilized to measure late drug pickup and medication possession. To ensure complete data collection, manual review of paper charts was conducted for all participants, and any data missing from Smartcare were entered into the electronic medical record system by study staff. Additionally, for intervention participants, group meeting attendance and symptom screening data were obtained through meeting attendance registers that were later entered electronically into a study database. Outcome ascertainment occurred 12 months post-enrollment and was accompanied by administration of a patient exit survey.

Qualitative interviews lasted approximately 45 minutes, while focus groups lasted 1–2 hours, and all were conducted in English or a local language (Nyanja, Bemba, or Tonga) according to the preference of the participants. At midline, semi-structured guides included questions about the introduction, enrollment, and implementation of the AC model. At endline, guides included questions on patient, provider, and study staff experiences with model implementation and the practicality of integration of these models into the current health system.

### Analysis

**Fig 1:**
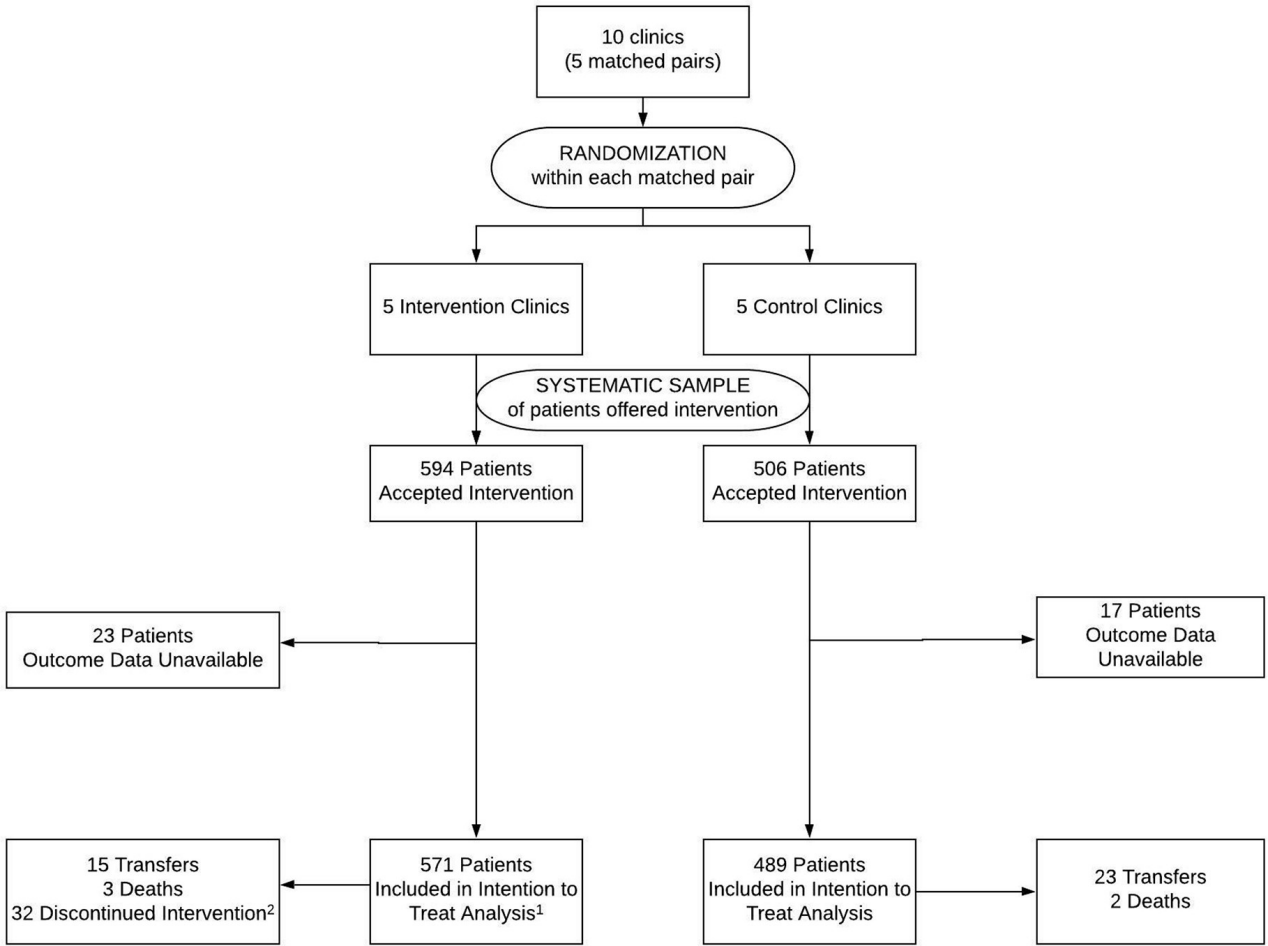
Participant flowchart. Eligible patients at intervention and control clinics were offered the intervention (i.e., assessed for willingness to participate in an adherence club), but only participants at intervention clinics received the intervention. ^1^Two patients who did not ultimately receive the intervention were still included in the intervention arm of the analysis as patients were analyzed independently of receiving the allocated treatment. ^2^Reasons for intervention discontinuation: pregnancy (*n =* 10), unable to be located >30 days after a missed adherence club meeting (*n =* 6), patient preference for facility-based care (*n =* 4), dismissed because of inability to follow adherence club rules (*n =* 4), diagnosed with tuberculosis (*n =* 4), and other (*n =* 4).

All patients who accepted AC participation at intervention and control facilities were analyzed independently of receiving the allocated treatment (Fig 1). The primary outcome was time to first late drug pickup (defined as greater than 7 days late) (S1 Text). Kaplan–Meier survival analyses were conducted to summarize time to first late drug pickup. The adjusted effect was estimated using a Cox proportional hazards model with a random effect for center [19]. Covariate selection for the model was based on inclusion of variables that were a priori considered to be potential confounders. These included age, sex, illness at care enrollment (defined as WHO stage III or IV or CD4 < 200 cells/mm^3^ at care enrollment), time since ART initiation, and MPR at study enrollment. In secondary analyses of the primary outcome, we explored longer intervals of lateness to define a missed pharmacy visit (14 and 30 days). All analyses were conducted with Stata version 15.0 (StataCorp, College Station, Texas, US).

Secondary outcomes included MPR and implementation outcomes as outlined by Proctor et al. [20]. MPR is defined as the proportion of time that a patient has ART in their possession over 12 months. Drug possession (assessed either via delays in drug pickup or MPR) is recognized as a reliable measure of adherence, with previous studies showing a strong negative association between MPR and viral load, treatment failure, and poor clinical outcomes [21–26]. For MPR calculation, we used drug pickup data in Smartcare to ascertain the number of days of ART non-possession in the year after study enrollment; MPR was calculated as 365 minus the number of days of ART non-possession, divided by 365, multiplied by 100 (to express MPR as a percentage). Approximately 2 to 3 extra days of medication are given at each drug pickup; therefore, in our analysis patients began to accrue medication non-possession 3 days after a missed drug pickup. Patients who died or transferred to another clinic were censored at the date of death or date of transfer, respectively.

Five main implementation outcomes (adoption, acceptability, appropriateness, feasibility, and fidelity) were analyzed quantitatively and qualitatively. Adoption (defined as “the intention, initial decision, or action to try or employ an innovation or evidence-based practice” [20]) was assessed quantitatively by describing the proportion of patients who accepted, joined, and attended AC meetings amongst those offered participation. Patient acceptability (defined as “the perception among implementation stakeholders that a given treatment, service, practice, or innovation is agreeable, palatable, or satisfactory” [20]), feasibility (defined as “the extent to which a new treatment, or an innovation, can be successfully used or carried out” [20]), and appropriateness (defined as “the perceived fit, relevance, or compatibility of the innovation for a given setting and to address a particular issue or problem” [20]) were assessed qualitatively. Fidelity of implementation (defined as “the degree to which an intervention was implemented as it was prescribed in the original protocol or as it was intended by the program developers” [20]) has 5 dimensions including “adherence, quality of delivery, program component differentiation, exposure to the intervention, and participant responsiveness or involvement” [20]. We analyzed the last 2 dimensions by examining the proportion of scheduled AC visits attended.

For qualitative analysis, a codebook was developed per a thematic framework [27] to evaluate acceptability, appropriateness, and feasibility. The concept of acceptability was used to synthesize individual stakeholders’ general experiences of, and feelings about, ACs. The concept of appropriateness was used to capture more specific assessments of the psychosocial or clinical implications of the AC, in particular the model’s ability to improve engagement in HIV care. The concept of feasibility was used to capture stakeholders’ perceptions regarding the material capacity (e.g., human resourcing, logistical capacity, drug availability, infrastructure) and organizational capacity (e.g., work culture and oversight) of the health centers and broader health system to support, scale up, and sustain the model. The lead qualitative investigator transformed transcripts into projects for coding in NVivo (QSR International, Doncaster, Australia). Five coders independently coded the same 2 transcripts to refine and validate an initial, deductively constructed codebook. Codes were then used to refine themes through inductive reasoning. In a second round, 3 coders independently coded 3 additional transcripts to achieve and confirm consistency in coding. With high inter-coder agreement these 3 coders subsequently coded the remaining transcripts independently.

Viral load suppression at 12 months was a planned secondary outcome but could not be evaluated due to unexpected inaccuracy of test results from dried blood spot (DBS) specimens (collected during study enrollment and exit procedures on all patients) and lack of availability of plasma viral load tests (collected in routine clinical care but available for less than 50% of study participants). Despite use of a validated DBS assay (COBAS AmpliPrep/COBAS TaqMan, Roche Diagnostics, Indianapolis, Indiana, US) [28–30] and detailed laboratory investigation, a clear cause for the assay error could not be determined, as was reported to the institutional review boards at the University of Zambia and University of California, San Francisco. Other secondary outcomes (S1 Text), including costs and cost-effectiveness, are planned for separate publication.

This trial is registered at ClinicalTrials.gov (NCT02776254). The trial protocol (S1 Text) and CONSORT checklist (S2 Table) are included for reference. Written informed consent was obtained from participants, and ethical approval was obtained from the institutional review boards at the University of Zambia and University of California, San Francisco.

## Results

### Patient characteristics

Among 571 intervention patients, median age was 42 years (IQR 35–48), 356 (62%) were female, median CD4 count at study enrollment was 512 cells/mm^3^ (IQR 327–653), and median time since ART initiation was 4.8 years (IQR 2.2–7.2) (Table 1). Baseline demographic characteristics were similar for the 489 control patients with respect to age, sex, time since ART initiation, CD4 at study enrollment, and baseline metrics of retention.

**Table 1:** Baseline characteristics of intervention and control participants.

Characteristics	Intervention *n =* 571	Control *n =* 489
Female sex	356 (62%)	322 (66%)
Median age (years)	42.0 (34.9–48.0)	40.8 (34.0–47.7)
Initial CD4 count^1^ (cells/mm^3^)	441 (271–565)	475 (306–631)
WHO stage at HIV care enrollment^2^		
Stage 1	215 (42%)	215 (50%)
Stage 2	129 (25%)	95 (22%)
Stage 3	153 (30%)	109 (25%)
Stage 4	13 (3%)	11 (3%)
WHO stage III or IV or CD4 < 200 cells/mm^3^ at HIV care enrollment^3^	159 (30%)	118 (27%)
Time since enrollment in HIV care (years)	5.2 (2.6–7.6)	5.6 (3.0–7.6)
Time since ART initiation^4^ (years)	4.8 (2.2–7.2)	5.0 (2.3–6.9)
CD4 count at study enrollment^5^ (cells/mm^3^)	506 (327–649)	533 (371–682)
0–100	17 (4%)	4 (1%)
101–200	14 (3%)	3 (1%)
201–350	98 (22%)	51 (17%)
351–500	100 (23%)	86 (29%)
>500	209 (48%)	148 (51%)
Medication possession ratio at study enrollment^6^ (%)	83 (76–95)	83 (78–91)
Late drug pickup (>7 days late) in year prior to study enrollment	305 (53%)	271 (55%)

Data are given as *n* (%) or median (IQR).

^1^Intervention: 565/571; control: 463/489.

^2^Intervention: 510/571; control: 430/489.

^3^Intervention: 525/571; control: 444/489.

^4^Intervention: 566/571; control: 485/489.

^5^Intervention: 439/571; control: 292/489.

^6^Intervention: 565/571; control: 482/489.

### Retention in care: Time to first late drug pickup

**Fig 2:**
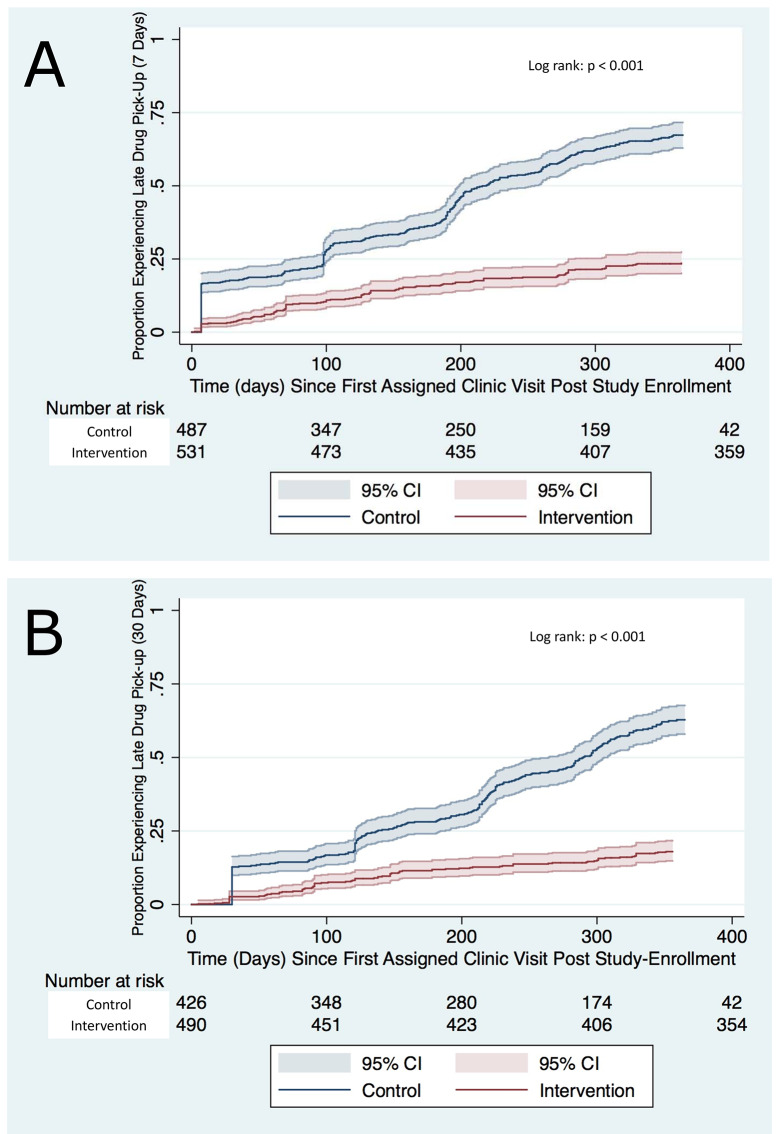
Time to first late drug pickup. Late drug pickup defined as >7 days late (A) or >30 days late (B).

Kaplan–Meier survival data for first late drug pickup (>7 days late) (Fig 2A) was examined at various time points. There was a statistically significant difference in time to first late drug pickup between the intervention and control groups (log rank test: *p* <0.001). Twelve-month cumulative incidence of first missed drug pickup was 0.24 (95% CI 0.20–0.27) in the intervention group and 0.67 (95% CI 0.63–0.72) in the control group (Fig 2A). The difference between groups persisted even when the interval of lateness was increased to 30 days (Fig 2B). Accounting for competing risk due to death gave similar results owing to the small number of deaths. In adjusted survival analyses, the rate of late drug pickup was lower in intervention participants compared to control participants (adjusted hazard ratio [aHR] 0.26, 95% CI 0.15–0.45, *p* < 0.001) (Table 2).

**Table 2:** Unadjusted and adjusted Cox proportional hazards model results of late drug pickup in intervention compared to control participants.

Predictor	Unadjusted hazard ratio (95% CI)	*p*-Value	Adjusted hazard ratio (95% CI)	*p*-Value
Intervention	0.26 (0.21–0.32)	<0.001	0.26 (0.15–0.45)	<0.001
Male sex	1.34 (1.11–1.61)	0.002	1.53 (1.24–1.88)	<0.001
Age at enrollment (per year)	0.99 (0.98–1.00)	0.019	1.00 (0.98–1.01)	0.41
Time since ART initiation (per year)	0.98 (0.95–1.00)	0.107	0.99 (0.95–1.03)	0.46
WHO stage III or IV or CD4 < 200 cells/mm^3^ at HIV care enrollment	0.87 (0.70–1.07)	0.193	0.96 (0.76–1.22)	0.76
Medication possession ratio (%) at study enrollment	0.99 (0.99–1.00)	0.016	1.00 (0.99–1.00)	0.31

Late drug pickups were more frequent in the control group. Among 489 control participants, 205 (42%) were more than 7 days late only 1 time, while 126 (25%) were late 2 or more times during the study period. In comparison, among the 569 patients who received the AC intervention, 105 (18%) were late only 1 time, and 81 (14%) were late 2 or more times. Although late drug pickups were more frequent in the control group, among those who did miss a drug pickup, time to return after first late drug pickup was similar in both groups: median of 25 days (IQR 5–63) in the intervention arm and 15 days (IQR 3–84) in the control arm (S1 Fig). This difference was not statistically significant (log rank test: *p* = 0.25).

### Adherence: MPR

**Fig 3:**
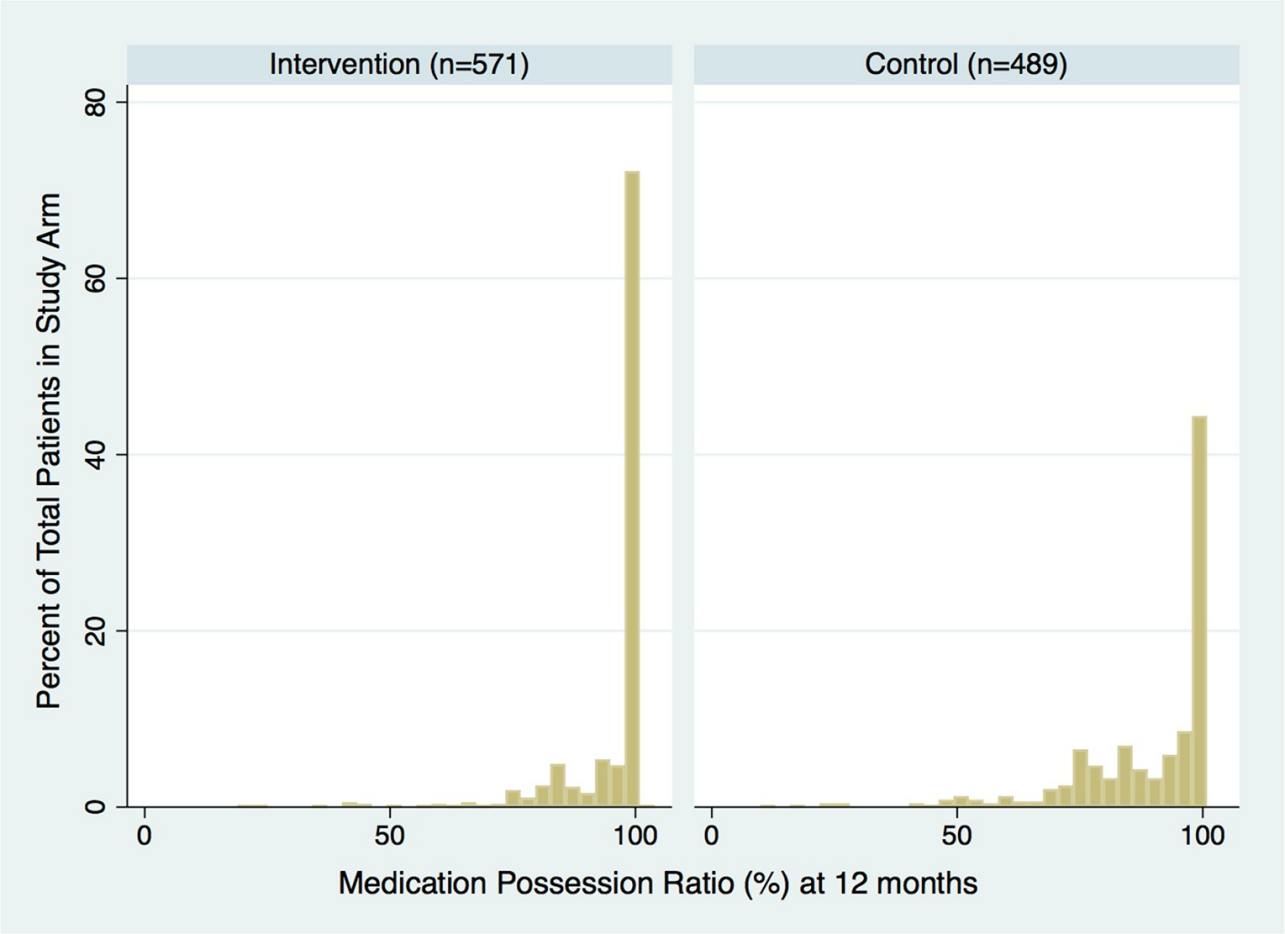
Twelve-month medication possession ratio (%) in intervention and control participants.

Baseline MPR at study enrollment was similar between treatment groups, at 83% (Table 1). Median MPR at 12 months post-enrollment was 100% (mean 95%; IQR 96%–100%) in the intervention arm compared to 96% (mean 89%; IQR 80%–100%) in the control arm (*p* < 0.001) (Fig 3).

### Adoption

**Fig 4:**
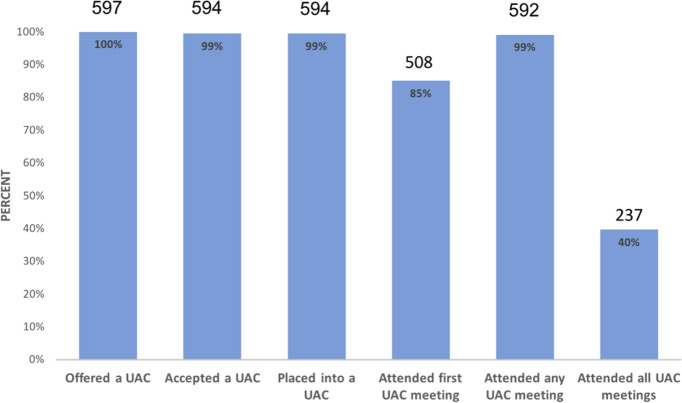
Individual patient uptake of adherence club model in Zambia. UAC, urban adherence club.

Of 597 patients offered AC participation, 594 (99%) accepted; 508 (85%) attended their first meeting (Fig 4), and 237 (40%) attended all AC meetings, with 194 (33%) missing 2 or more group meetings.

### Acceptability

Table 3 summarizes key qualitative themes relating to acceptability, appropriateness, and feasibility synthesized from the data. Patients who participated in focus group discussions described ACs as being highly acceptable as a result of more time during normal working hours to address livelihood concerns regardless of employment status (“We spend less time here and within a short time we get back, that is very beneficial.”—female participant, Petauke); reduced concerns about stigma due to decreased exposure during the facilities’ busiest hours; and reduced self-stigma and improved access to information and group support (“We encourage each other when we sit and we also teach others how to take drugs…If we have seen that our friends are in problems, we try by all means to encourage each other.”—male participant, Petauke). Healthcare workers too found the model highly acceptable due to perceptions of reduced clinic congestion and, in select cases, workload; as one participant described: “Before the model, the congestion [was high]…Maybe the doctor was tired and stopped doing clinical work; even adherence counseling…was hectic. But because of this model…it was less.”

**Table 3:** Key qualitative research findings evaluating patient and healthcare worker perspectives on intervention acceptability, appropriateness, and feasibility.

Outcome	Patient perspectives	HCW perspectives
**Acceptability**	• More time during normal working hours to address livelihood and other family responsibilities (described variously by patients regardless of employment status) • Reduced concerns about stigma due to reduced and more convenient time being spent at clinic (less visibility at clinic) • Reduced self-stigma • Improved access to information and group support	• Reduced clinic congestion and, in select cases, workload
**Appropriateness**	• Reduced stress and logistics in accessing medication • Preference for group counseling over one-on-one adherence counseling due to having more time to ask questions, sharing own experiences, and learning from others’ experiences • Patient-centered approach afforded respect and responsiveness • Mixed gender groups felt to be appropriate • Lack of inclusion of children in ACs felt inappropriate for some mothers as they still had to accompany their children to the facility for all their visits	• Intervention aligns with existing clinical guidelines • One-on-one counseling more effective and appropriate • Members should be required to be on ART for 12 months (instead of 6) and/or they should have more frequent clinical checks
**Feasibility**	• Patients were offered a variety of time slots (more patients opted for weekend meetings; time conflicts with church were not an issue as previously anticipated) • Supportive attitude and responsiveness of AC coordinators should be maintained if government takes over AC services	• Lay HCW important resource, and government employment of lay HCW needed • Increased human resources for health needed in key areas, most notably pharmacy

AC, adherence club; HCW, health care worker.

### Appropriateness

Qualitative findings (Table 3) highlighted that ACs were felt to be appropriate by patients because of reduced stress and logistics in accessing medication, preference for group counseling over one-on-one counseling, and strengthened patient-centered approach, where patients had “an opportunity to interact with the staff” without fear that health workers would be tired, moody, or disrespectful. Group size and mixed gender groups were generally felt to be appropriate; however, several mothers felt that children should be included in AC groups as parents were otherwise required to accompany their children to all facility visits. Health workers were more equivocal, with some acknowledging the pragmatic advantages of ACs but others expressing concern about the importance of retaining “more personalized” (one-on-one) counseling and more frequent clinical checks.

### Feasibility

Both patients and healthcare workers described the AC model as being feasible assuming sufficient staff and funding were available. Almost all participants stressed that ACs would only be successfully integrated and scaled in the current health system if (1) government-employed AC group leaders were selected and trained to be respectful and patient-centered by being “friendly” and “helpful” and to “talk nicely,” instead of “always chatting” and “shouting at us”; (2) there is formal employment of lay healthcare workers, who play a key role in AC functions (“These lay counsellors or community health workers play a major role…The government should even employ them.”—healthcare worker, Lusaka); and (3) there are increased human resources for health, particularly to meet pharmacy needs (“If it is to be handed to government health workers, I think they should just increase man power so that there are enough workers at the facility.”—male participant, Lusaka).

### Fidelity

**Fig 5:**
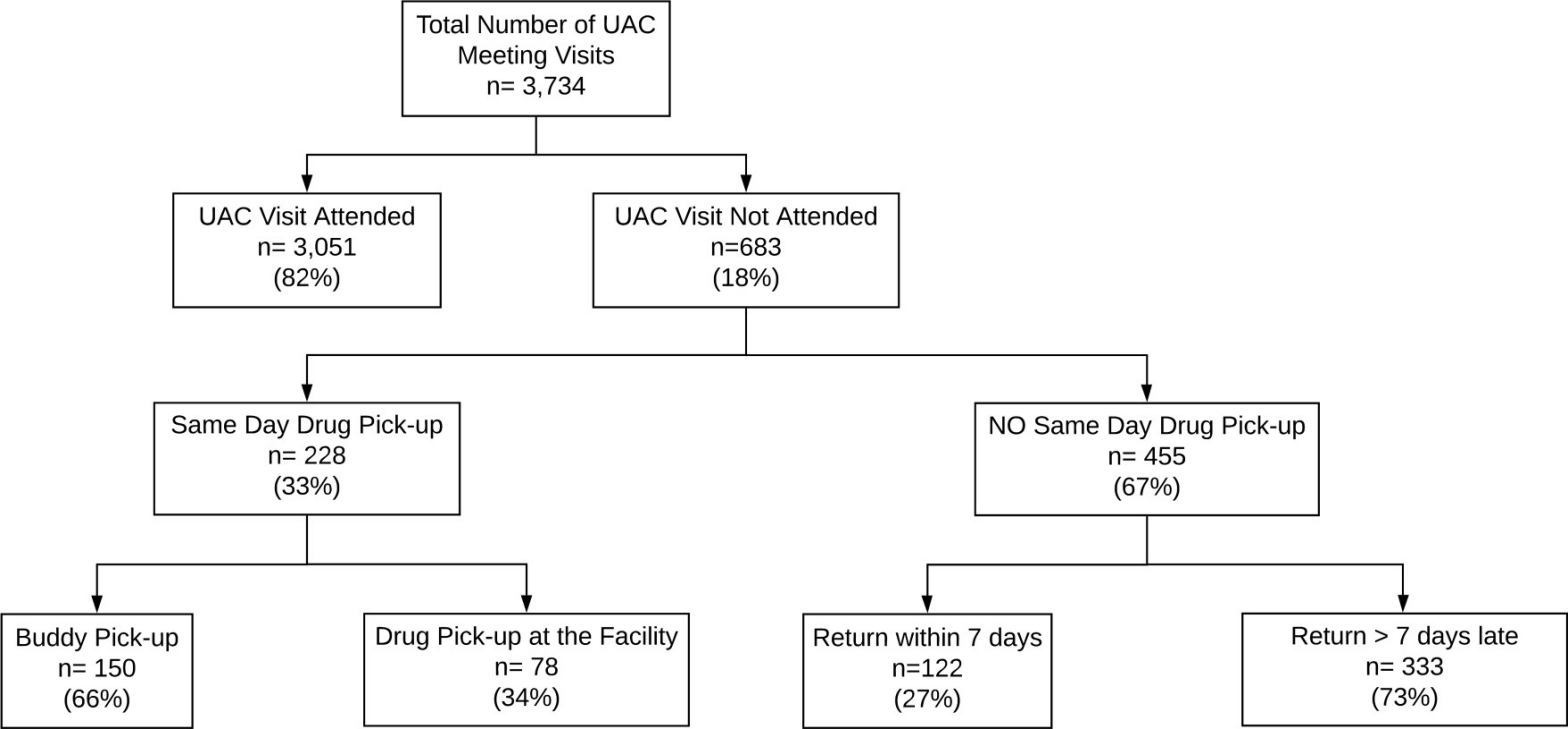
Meeting attendance and on-time versus delayed drug pickup. UAC, urban adherence club.

Assigned appointment frequency for drug pickup in the intervention group coincided with AC meetings and was every 2 months in the first 6 months of the study period and then every 3 months in the second 6 months of the study period. In comparison, the median drug pickup appointment interval was 90 days (IQR 60–92 days) over the 12-month study period in the control group. Overall, of 3,734 scheduled AC meeting visits, 683 (18%) were not attended. However, drug pickup within 7 days still occurred for 350 (51%) of these missed visits, either via buddy pickup or early return for drug pickup at the facility (Fig 5). Intervention discontinuation occurred among 32 participants, with pregnancy being the most common reason for discontinuation (*n =* 10) (Fig 1).

## Discussion

We found that participation in an AC model was associated with a 74% reduction in risk of experiencing a late drug pickup, which is strongly associated with retention and is a key driver of virological rebound [22]. Similar effects were observed when the definition of lateness was extended from 7 to 30 days. These effects were observed despite the fact that nearly 20% of scheduled group meeting visits were not attended. Our findings suggest that although participants did not always adhere to group-based ART delivery, model participation did facilitate alternative methods of on-time drug pickup (via buddy system or early return to clinic after a missed AC visit). Although intervention and control participants returned to the clinic at similar rates after missing a drug pickup, fewer overall missed drug pickups, combined with early return, accounted for a median MPR of 100% (no gaps in medication possession) among intervention participants.

Our study findings are consistent with those of earlier published observational studies showing high retention in ART care among patients enrolled in ACs and is the first to our knowledge to provide non-observational effectiveness data that support this intervention. Previously published observational studies in South Africa [11,13] reported high retention in ART care, defined as any contact with club or facility, as their primary study outcome. While they did not detail club meeting attendance, one of the studies did note that 27% of patients sent a buddy to at least 1 club visit. More recently, a pragmatic trial conducted in South Africa (comparing 24-month retention in clinic-based versus community-based ACs) found that missing a club visit was common regardless of club type [31]. Twenty-six percent of patients in the South African cohort missed drug pickup completely after missing a club visit; however, only 10% were lost from ART care. In our study, although nearly 20% of scheduled club visits were missed, missing drug pickup completely (no buddy pickup and no clinic return within 7 days of a missed club visit) occurred in only 10% of scheduled visits overall. Thus, collectively, these studies appear to share several findings: Retention in ART care is high among patients who initially accept club participation (even after club discontinuation), individual club meeting attendance is not uniformly high, and patients commonly utilize the flexibility afforded by the buddy system and drug pickup at the facility.

Frequent alternative drug pickup, as found in our study, underscores two key points of public health significance: the need for DSD models to remain flexible and patient-centered if they are to be effective and the need to reconsider the settings in which ACs afford greater benefits compared to other popular forms of DSD such as visit spacing and streamlined facility-based drug delivery alone. In the pragmatic trial conducted by Hanrahan et al. [31], patients were removed from the club and referred back to facility-based care if they sent a buddy to 2 consecutive meetings, had two late medication pickups at the facility, or missed a medication pickup entirely (no buddy and no late medication pickup). Loss to club-based care was high in both community- and clinic-based clubs (nearly 50%), and involuntary discontinuation for missed/late visits was the most common reason. Similar policies exist in Western Cape Province in South Africa, where ACs have been broadly scaled up, with greater than 50%–70% of eligible patients enrolled in clubs to date [12,32]. Qualitative work from this setting revealed that club expulsion for missed club meetings was seen as an unfair punishment and was associated with a breakdown of trust between the patient and healthcare system [33]. In patients for whom access issues limit on-time attendance to club meetings, requiring increased contact with the facility may only make the situation worse [34]. Given that a key element of DSD is increased patient-centeredness, rigid and “one size fits all” requirements within DSD models are unlikely to be successful, just as they proved to be suboptimal in maintaining long-term care engagement in facility-based care models [35]. Rather, individual reasons for missed club visits should be elicited and patient-specific responses developed prior to considering club expulsion. It is also evident, given the number of patients that became pregnant during study follow-up, that flexibility for pregnant patients or others with changing clinical needs who may temporarily require more facility contact (i.e., for tuberculosis treatment) is also necessary.

Given the theoretical complexities and resources required to assemble and maintain ACs and the relatively common occurrence of missed club visits, program developers and policymakers may appropriately question what benefits a group-based ART delivery model affords over improved individual models of care (visit spacing and streamlined drug pickup at the facility). ACs exist on a continuum between other models such as community adherence groups (in which small groups [e.g., 6] of patients meet monthly in their homes and rotate drug pickup responsibilities for the entire group) and fast-track drug pickup (in which individuals receive longer duration drug refills and expedited drug pickup at the facility) [36]. However, the mechanism of effect for each of these models is poorly understood and likely multifactorial [37,38]. Theoretical behavioral mechanisms by which ACs improve retention in ART care include greater self-efficacy, perceived social support, empowerment, and perceived benefits [38]. The group format of urban ACs theoretically affords greater peer social support than fast-track drug delivery alone. ACs may also offer relative advantages over fast-track refills for patients who are unable to visit the clinic during regular working hours due to employment. Both of these mechanisms were supported by our qualitative research results but need to be elucidated further. It is unclear if these constitute important mechanisms of effect or whether expedited drug refills and/or “VIP treatment” at the facility due to club participation played a larger role in retaining patients in care. Comparative effectiveness studies, comparing more complex interventions (i.e., urban ACs and community adherence groups) to visit spacing and fast-track refills alone, and seeking in-depth understanding of contextual influences on efficacy, are urgently needed.

There were several limitations to our study. While matching of clusters (in this case, facilities) helped improve the similarity between treatment arms at baseline (particularly given the small number of clusters), comparability is limited to the characteristics that were matched upon. The principle of intention to treat is also challenged in cluster RCTs because of the lack of a statistical method to handle non-recruited participants. Furthermore, patients within each facility were invited to participate through a systematic selection process, which falls short of actual randomization. Despite these limitations, we attempted to minimize bias and confounding by matching on the baseline value of the endpoint of interest, performing additional adjustment during analysis, and analyzing patients independently of receipt of the allocated treatment [39]. Additionally, we lacked accurate viral load data in over 50% of the patients and were therefore unable to include these data in our secondary analyses. Duration of follow-up in our study was relatively short (12 months), and therefore we have limited ability to comment on sustainability and long-term outcomes of the intervention.

As DSD models are being scaled up throughout sub-Saharan Africa, our study provides timely and robust evidence of the effectiveness of the AC intervention. Although visit spacing and group-based ART drug pickup and counseling are core components of the AC intervention, our study highlighted that flexible drug pickup by a buddy and/or late drug pickup at the facility without punitive repercussions was also an essential part of the intervention. However, more work needs to be done to understand the causal impact and resource cost of the AC intervention compared to simpler non-group-based DSD interventions (e.g., visit spacing and fast-track refills) to optimize the quality and efficiency of a DSD-based health system that could eventually provide not only ART, but medicines for other chronic diseases.

## Supporting information

S1 Fig Time to return to clinic after first missed drug pickup in intervention and control participants. (TIFF)

S1 Table Sample size calculation table. (DOCX)

S2 Table CONSORT checklist. (DOCX)

S1 Text Study protocol. (DOCX)

## Abbreviations

AC: adherence club
aHR: adjusted hazard ratio
ART: antiretroviral therapy
DSD: differentiated service delivery
MPR: medication possession ratio
