# Supplementary figures and images for: Participation in adherence clubs and on-time drug pickup among HIV-infected adults in Zambia: A matched-pair cluster randomized trial
Source: PLoS Med. 2020 Jul 1;17(7):e1003116. doi: 10.1371/journal.pmed.1003116 (PMC7329062; doi:10.1371/journal.pmed.1003116)

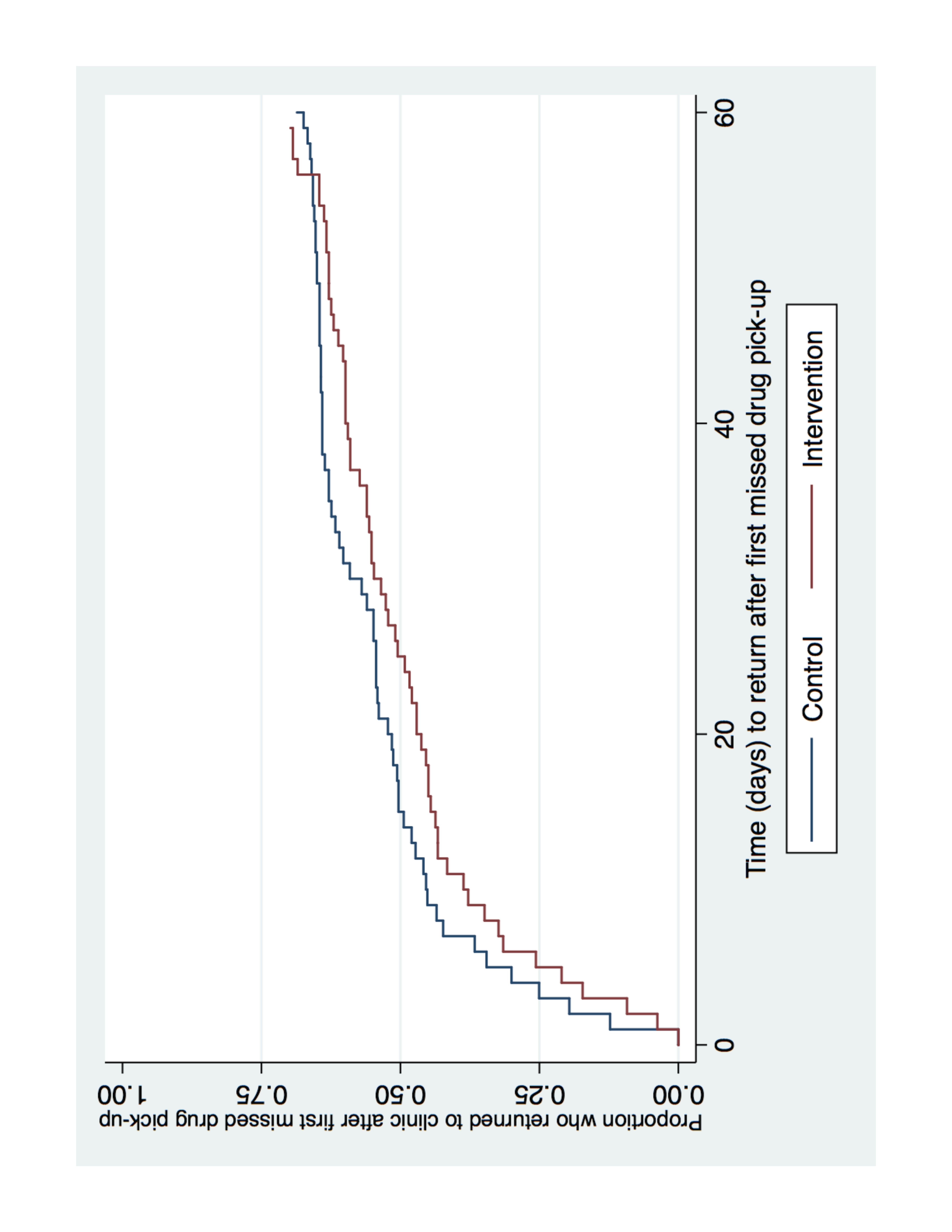

Supplement: S1 Fig — (TIFF) [file pmed.1003116.s001.tiff]
