# Supplementary material for: Participation in adherence clubs and on-time drug pickup among HIV-infected adults in Zambia: A matched-pair cluster randomized trial
Source: PLoS Med. 2020 Jul 1;17(7):e1003116. doi: 10.1371/journal.pmed.1003116 (PMC7329062; doi:10.1371/journal.pmed.1003116)
Supplement: S1 Table — (DOCX) [file pmed.1003116.s002.docx]

| alpha | Pi0 | Pi1 | K | J | N | Power |
| --- | --- | --- | --- | --- | --- | --- |
| **Modify Pi0 and Pi1** |  |  |  |  |  |  |
| 0.05 | **0.8** | **0.6** | 0.1 | 30 | 5 | 0.62 |
| 0.05 | **0.8** | **0.4** | 0.1 | 30 | 5 | 0.997 |
| 0.05 | **0.7** | **0.5** | 0.1 | 30 | 5 | 0.63 |
| 0.05 | **0.6** | **0.4** | 0.1 | 30 | 5 | 0.66 |
| 0.05 | **0.6** | **0.3** | 0.1 | 30 | 5 | 0.96 |
| 0.05 | **0.4** | **0.2** | 0.1 | 30 | 5 | 0.7989 |
|  |  |  |  |  |  |  |
| **Modify k** |  |  |  |  |  |  |
| 0.05 | 0.6 | 0.3 | **0.1** | 30 | 5 | 0.96 |
| 0.05 | 0.6 | 0.3 | **0.2** | 30 | 5 | 0.82 |
| 0.05 | 0.6 | 0.3 | **0.3** | 30 | 5 | 0.597 |
|  |  |  |  |  |  |  |
| **Modify j** |  |  |  |  |  |  |
| 0.05 | 0.6 | 0.3 | 0.1 | **10** | 5 | 0.64 |
| 0.05 | 0.6 | 0.3 | 0.1 | **15** | 5 | 0.7988 |
| 0.05 | 0.6 | 0.3 | 0.1 | **30** | 5 | 0.96 |
| 0.05 | 0.6 | 0.3 | 0.1 | **45** | 5 | 0.991 |
| 0.05 | 0.6 | 0.3 | 0.1 | **90** | 5 | 0.999 |
| 0.05 | 0.6 | 0.3 | 0.1 | **120** | 5 | 0.999 |
|  |  |  |  |  |  |  |
| **Modify n** |  |  |  |  |  |  |
| 0.05 | 0.6 | 0.3 | 0.1 | 30 | **5** | 0.96 |
| 0.05 | 0.6 | 0.3 | 0.1 | 30 | **6** | 0.99 |
| 0.05 | 0.6 | 0.3 | 0.1 | 30 | **7** | 0.997 |

alpha: significance level - usually assumed to be 5%

Pi0: average proportion experiencing event at 12 months for control clinics

Pi1: average proportion experiencing event at 12 months for intervention clinics

k: matched pair coefficient of variation

j: number of people per clinic

n: number of clinics per arm
