# Supplementary material for: Participation in adherence clubs and on-time drug pickup among HIV-infected adults in Zambia: A matched-pair cluster randomized trial
Source: PLoS Med. 2020 Jul 1;17(7):e1003116. doi: 10.1371/journal.pmed.1003116 (PMC7329062; doi:10.1371/journal.pmed.1003116)
Supplement: S1 Text — (DOCX) [file pmed.1003116.s004.docx]

**Community ART for Retention in Zambia:**

**Evaluating the Feasibility, Effectiveness, and Efficiency of Decentralized and Streamlined Antiretroviral Therapy Care Models**

**(Community ART)**

**Protocol Version 4.0**

**13 April 2016**

Table of Contents

1.0 Background and Introduction 4

1.1 Rationale for study 4

1.2 Significance 4

1.3 Aims 5

1.4 Specific Objectives 5

1.5 Audience and Stakeholder Participation 5

1.6 Ethical Issues 6

2.0 Literature Review 6

3.0 Methodology 7

3.1 Objective 1: To determine the acceptability, appropriateness, and feasibility of elements of a differentiated care system in Zambia. 8

3.1.1 Methodology for Objective 1 8

3.1.2 Population 8

3.1.3 Measurements and Procedures 8

3.1.4 Analytic Approach 9

3.1.5 Sampling Approach/Sample Size Considerations 9

3.2 Objective 2: To evaluate the effectiveness, efficiency, and health care quality of a differentiated system of care that includes targeted models of care. 10

3.2.1 Methodology for Objective 2 10

3.2.2 Population 11

3.2.3 Procedures 11

3.2.4 Measurements 13

3.2.5 Analytic Approach and Sample Size Considerations 14

3.2.6 Study Timeline 16

3.3 Objective 3: Methodological Toolkit 16

3.4 Considerations for Human Participants 16

3.4.1 Informed Consent 16

3.4.2 Risks to Participants 17

3.4.3 Methods to Minimize Risks 18

3.4.4 Anticipated Benefits to Participants 18

3.4.5 Privacy of Individuals 18

3.4.6 Confidentiality of Data 18

3.4.7 Study Discontinuation 19

3.5 Data Usage 19

4.0 Budget 20

5.0 Time Frame 20

6.0 Reference List 21

7.0 Appendices 22

7.1 Appendix 1: Selected Differentiated Care Model Outcome Studies 22

7.2 Appendix 2: Outcomes Framework 24

7.3 Appendix 3: Objective 1 Overview 25

7. 4 Appendix 4: Overview of four models of differentiated care implemented in Objective 2 27

7.5 Appendix 5: Routine schedule of activities within four models of differentiated care implemented in Objective 2 28

7.6 Appendix 6: Power calculations for Objective 2: CAG and UAG 29

# Background and Introduction

Although the scale up of antiretroviral therapy (ART) in Zambia has reached over 600,000 adults and children, ^1^ the present health care systems is neither optimally efficient nor effective. To improve both the effectiveness and efficiency of HIV care delivery from both the patient and health system perspectives, we plan to evaluate the local preferences for, and implementation of, “differentiated” delivery strategies that reduce unnecessary contact with facility-based care and improve community support for stable patients in rural and urban areas and low and high patient volume sites.

We hypothesize that differentiated care approaches will be more effective and more efficient as measured by improved patient retention in care, reduced clinic congestion, and provider and patient satisfaction. ^2-13^ Although implementers have piloted some of these strategies elsewhere, there are a dearth of examples in which they have been taken to scale, and data on their outcomes are lacking from less resourced countries outside of South Africa. Our implementation science study uses a variety of methodologies to both understand preferences and acceptability of new models of care by patients and their family members, healthcare workers (HCW), policy makers and other key stakeholders, and to rigorously, yet pragmatically produce unbiased assessments of the magnitude of benefits to the health system and to patients, along with a rich local understanding of implementation processes.

## 1.1 Rationale for study

**Continued scale up of ART using traditional facility-based ART delivery methods is likely to compound human resource and infrastructure deficiencies.** Although over 600,000 in Zambia have started treatment, acceleration of case identification as well as broadening treatment criteria mean that expected numbers may double in the next five years. “First-generation” ART services tended to be facility-based, staffed by higher-level HCW and characterized by inflexible patient appointment schedules, short visit intervals, and administrative requirements (e.g. standardized care and treatment counselling for all patients, etc). This service delivery model has persisted despite the fact that an estimated 50% of patients are stable on ART with no complaints. ^1^ This “one-size-fits-all” approach contributed to national ART retention rates of 67.4% at 24 months in 2011.

**Differentiated care – including fuller engagement of the community – may be a more effective and efficient service delivery model.** Innovative delivery strategies have begun to move away from facility to community based services. Community Adherence Groups, which emerged first in Mozambique, are patient groups of six stable individuals who take turns attending the clinic, thereby reducing the number of visits from 6-12 for each patient, to 2 each over the course of a year. This markedly reduces patient transportation burden, leverages social ties to heighten adherence to the clinic visit, and simultaneously reduces clinic volume and congestion, thus allowing providers to focus on the less stable patients who remain in the facility based model of care. While seemingly effective, issues such as selection bias, counterfactual construction, loss to follow-up, and use of viral load have not been addressed in a compelling fashion. This study will address these issues and establish the data that the Government of the Republic of Zambia will need to scale these new models nationally.

## 1.2 Significance

The existing health delivery systems for HIV infected patients are not optimal: loss to follow up exceeds 25% at one year and 33% at two years, thus threatening the gains of widespread ART roll out. ^14^ Opportunity costs for clinic attendance are high - on average, patients must travel long distances to access care and may lose an entire day of work productivity. In addition, burnt out health care workers and clinic congestion can make a clinic environment unpalatable for patients. Finally, psychological barriers, including stigma and depression, are common and can undermine motivation to continue care. These factors are all exacerbated by a one-size-fits all health delivery system and excessive demands on the patient.

Health systems are overburdened and complicated delivery algorithms tie up essential resources. A recent US Government technical review of the HIV program in Zambia recognized the serious infrastructural challenges within the current health system and the promise of care decentralized into the community as a tool to decongest overcrowded sites: *“It is clear that the public health system has already surpassed the capacity of its human resources and physical infrastructure to address the rapidly growing number of ART clients.  Although substantial improvements can be made working through the existing system, community-based models should be explored and supported by PEPFAR.”* Our goal is support the national system in Zambia to unlock existing capacity and target it toward those in need.

##

## 1.3 Aims

Our study seeks to create generalizable knowledge about the implementation process as well as the effectiveness and efficiency of a differentiated care system, by measuring patient health outcomes and implementation outcomes such as acceptability, feasibility, fidelity, and costs.

## 1.4 Specific Objectives

1.4.1 Objective 1:

**To determine the acceptability, appropriateness, and feasibility of a differentiated care system in Zambia**, by assessing perspectives of three key groups (1) patients and family members (2) health care workers and (3) government and local leaders.

1.4.1 Objective 2:

**To evaluate the effectiveness, efficiency, and health care quality of a differentiated care system that includes targeted models of care.**

Effectiveness of differentiated models of care will be assessed through retention in care, efficiency through cost-effectiveness analysis, and quality through qualitative and quantitative methods.

1.4.3 Objective 3:

**To develop a “methodologic” toolkit for assessment of local needs and preferences and for implementation during scale-up of differentiated care models in this and in other contexts**, using data from Objectives 1 and 2.

## 1.5 Audience and Stakeholder Participation

This study is a joint endeavor of the Zambian Ministry of Health (MoH), Ministry of Community Development, Mother & Child Health (MCDMCH), Bill & Melinda Gates Foundation (BMGF), and the Centre for Infectious Disease Research in Zambia (CIDRZ). Stakeholders include people living with HIV particularly the civil society organizations actively working with this population, community leaders and ordinary members in study sites, the Government of the Republic of Zambia (particularly health related line ministries and corresponding provincial, district and facility structures), the Centers for Disease Control and Prevention (CDC) in Zambia and other partners involved in HIV prevention, care and treatment in Zambia, other donors, and the men and women of Zambia. We will work closely with all stakeholders to ensure that data from this study is appreciated and understood. Where necessary, we will work with relevant technical working groups within the Ministries to adapt or develop policies to ensure that these findings are concretely translated into policy.

## 1.6 Ethical Issues

Overall, this study seeks to evaluate patient, provider, and policy maker perspectives as well as systems interventions that target health care facilities. In all instances the intervention is delivered at the clinic level but we will obtain written informed consent among both intervention and control participants. CIDRZ will work with control sites and the MOH to develop a feasible plan for offering the intervention at control sites after the study period. Such plans will take into account any changes to MOH policies, the CIDRZ funding environment, and other external factors.

For all four models, eligible patients within the intervention clinic will be offered enrollment in a systematic fashion until the site reaches the enrollment cap (see Appendix 4). Since it is expected that enrollment in the study will decrease the number of times a participant has to attend clinic (thus decreasing costs to the participant), transportation refunds will not be offered for participation. Patients who decline to consent and patients who do not meet eligibility criteria will receive standard of care at the intervention site. After the enrollment cap has been reached, the remaining patients at the intervention clinics will continue to receive the standard of care. At the time that informed consent is obtained, patients will be notified that the intervention is scheduled to last only through Month 25 of the study. CIDRZ will work with sites to create exit plans for the post-study period. Such plans will take into account any changes to MOH policies, the CIDRZ funding environment, and other external factors.

Additionally, in Objective 1, we use quantitative surveys and qualitative interviews of multiple stakeholders to understand needs and preferences. We will seek written informed consent to carry out these assessments.

This protocol, the informed consent documents, and any subsequent modifications will be reviewed and approved by the UNZA Biomedical Research Ethics Committee (BREC) and the necessary IRB’s responsible for oversight of the study. Further information on participant risks, benefits and other considerations for human participants are addressed in Section 3.5 of the protocol ‘Considerations for Human Participants’.

In each of these settings, the interviewer will provide verbal information about the study including the objectives of the study, the potential benefits and risks, and confidentiality. An information sheet, mirroring the information, which was provided verbally, will then be given to and retained by the participant. Both parties will sign a consent form. In the case of a participant being illiterate, an independent witness will additionally sign the consent form to confirm that the information provided verbally matches the written information. Privacy of individuals and confidentiality of data is detailed in Section 3.5.

# Literature Review

**The outcomes of patients in community adherence groups (CAGs) have been quantified, but the effects of a CAG as compared to patients continuing routine care has not been directly evaluated.** Data on community adherence groups (CAGs) is observational and drawn primarily from a single-arm study conducted in Mozambique. ^7,8^ Médecins Sans Frontières (MSF) pioneered the creation of CAGs ^15^ and found retention of 97.5% at a median follow-up time of 12.9 months and four-year retention rates of 92%. These studies, however, did not account for the fact that patients who are stable are selected for in these groups and there is no comparator group of comparable “stable” patients who did not join any of the groups. Rasschaert et al employed a mixed-methods approach to evaluate implementation of the CAG model in Tete, Mozambique. ^7,16^ Their evaluation provides a logic framework for the CAG intervention but does not quantify effects directly against a counterfactual.

**There is only one observational study that has evaluated urban adherence groups (UAGs) that was conducted in a township in Cape Town, South Africa.** ^6^ The clubs, consisting of individuals stable on ART for 12 months, met for 2 hours every 2 months and were facilitated by peer educators/lay counsellors who distributed pre-packaged ART and providing basic clinical consultation. This was a cohort study and included contemporaneous control patients receiving routine nurse-led care. The primary outcome was death or loss to follow-up. The secondary outcome was virologic rebound in patients supressed at study entry. At the end of the evaluation period (approximately four years), 97% of patients remained in care compared to 85% of control patients. After adjustment, participation was associated with a 57% reduction in loss to follow-up and 67% reduction in virologic rebound. There was a limited attempt at accounting for selection bias using inverse probability weighting.

**Streamlined pharmacy pick-up has been evaluated in Uganda where a cost-effectiveness analysis was conducted to compare a pharmacy-only refill program to the standard of care.**^11^ There was a non-significant decrease in odds of having a CD4 > 500 at the end of the study period with the intervention. Costs were calculated from a limited societal perspective and the incremental cost-effectiveness ratio (ICER) comparing intervention to standard of care was US $13,500 per patient with a CD4 > 500. There are no additional effectiveness studies evaluating this model of care, and none with the more comprehensive design of our proposed FAST-TRACK model.

**Although there are several studies using POC CD4 technology, few have evaluated it in the context of the HIV clinical care cascade or evaluated its effect on reducing time to ART initiation as part of a streamlined algorithm of care (such as START).**

Present day CD4 technology burdens systems and patients: at lower level facilities patients or specimens must be transported long distances. Repeatability of POC CD4 testing is excellent even with non-laboratory operators and field studies demonstrate feasibility and technical validity in program settings in Africa. No published studies have evaluated the use of a streamlined ART initiation program using POC CD4 testing.

A summary of select literature on the outcomes of differentiated care models is available in Appendix 1.

# Methodology

This proposal seeks to evaluate four models of streamlined HIV care that reduce the substantial time and cost currently incurred by patients seeking care. These four models are (1) community adherence groups (CAG), (2) urban adherence groups (UAG), (3) Fast-Track clinic visits and (4) streamlined ART initiation (START). We assess the first two models (CAG and UAG) using a cluster-randomized design because existing observational data already exist. The second two models (Fast-Track and START), for which there is little to no observational or implementation data, will be evaluated using an observational approach, in which selected clinics will implement the model and changes in the outcome will be compared to changes in other clinics not implementing the model through a difference in difference approach. Overall these four models enable greater effectiveness and efficiency of care delivery through tailoring delivery approach to patient needs.

## 3.1 Objective 1: To determine the acceptability, appropriateness, and feasibility of elements of a differentiated care system in Zambia.

### 3.1.1 Methodology for Objective 1

A mixed methods approach will be used to qualitatively and quantitatively evaluate perceptions regarding current challenges in accessing and adhering to life-long HIV treatment and to assess needs and preferences for various potential differentiated care models. We will assess perspectives from three key groups: (1) individual patients and family members (demand side) (2) health facilities –professional and community health workers (supply side) (3) government and local leaders. Four types of cross-sectional data collection methods will be utilized: in-depth interviews, focus group discussions, surveys, and discrete choice surveys. These will form the basis for sequential analysis of supply and demand side factors that may influence the uptake of specific differentiated care models.

### 3.1.2 Population

Critical government (MoH, MCDMCH) and non-government stakeholders involved in the delivery of HIV or other relevant community based services will be targeted for participation. From MoH we will seek participation from Directors (or their delegated representatives) from the Directorate of Clinical Care and Diagnostic Services and Directorate of Policy and Planning in addition to the National ART Program Coordinator. From MCDMCH we will seek interviews with the Permanent Secretary (or delegated representative), Monitoring and Evaluation officer, and the District Medical Officers in all areas where the study takes place. We will also interview both professional and lay health workers presently working under the ART program at the facilities. Pre-ART and patients on ART will also be included. To determine family preferences, we will include a sample of persons living in the same household as a current ART or pre-ART patient registered at a health facility selected for participation.

### 3.1.3 Measurements and Procedures

For Objective 1 we will utilize a combination of: In-Depth Interviews (IDIs), focus group discussions (FGDs), medical chart reviews, discrete choice experiments and a general survey.

*IDI:* Interviewers will utilize semi-structured question guides that are developed based on a review of regional literature and investigator experience and will be specific to the type of participant. Participants will be interviewed for approximately 1 hour and interviews will be conducted in English or a local language (Nyanja, Bemba, or Tonga). Where permission is granted, interviews will be audio-recorded for later transcription and analysis. Participants will be given travel reimbursement (ZMK50, equivalent to ~$7) if participation in the interview required them to travel.

*FGDs:* These will utilize semi-structured question guides that are developed based on a review of regional literature and investigator experience and will be specific to the type of participant. FGDs will last for 1-2 hours and will be conducted in English or a local language (Nyanja, Bemba, or Tonga) based on participant preference. All discussions will be facilitated by a trained discussant fluent in the local language and in English and, where permission is granted, discussions will be audio-recorded for later translation, transcription, and analysis. The discussant will provide ground rules for the discussion before beginning and will be responsible for re-directing the conversation if it goes off-topic. Participants will receive a nominal travel reimbursement (ZMK50 equivalent to ~$7).

*General survey:* We will utilize a cross-sectional survey to measure patient access and psychosocial needs. Access needs will be assessed with questions that address the following: appropriateness of current clinic operating hours; distance, cost, and time required to get to clinic; household income, assets, and expenditures; and opportunity costs of a clinic visit. We will carefully elicit patient costs on a comprehensive set of items related to health care utilization, including transport, communication, and food costs. To assess psychosocial needs we will apply previously validated scales and indices to measure stigma (Revised Berger), depression (PHQ-9), alcohol consumption (AUDIT-C), and domestic violence. The survey will take approximately 30-45 minutes to complete and will be administered by trained survey enumerators using tablets. The survey enumerator will read each survey question and all possible answers choices. Surveys will be conducted in the local language or English, based on the participant’s choice. Survey will initially be written in English, translated into the local language (including Nyanja, Bemba and Tonga) and then back- translated into English to ensure accuracy. Survey responses will be entered directly into the tablet by the survey enumerator and at survey completion all responses will be uploaded to the central database.

*Medical record review:* We will utilize the existing electronic clinical information system (SmartCare) to evaluate patient clinical needs. We will assess distribution of clinical needs over several dimensions: 1) degree of immunosuppression at entry into care (CD4, WHO Stage), 2) frequency of opportunistic infections (OIs) after treatment initiation, and 3) frequency of “ill” versus “healthy” visits.

*Discrete choice survey:* We will use Discrete Choice Surveys (DCSs) to assess patient preference for different, discrete characteristics of various models of community-based ART delivery. We will select key attributes (or aspects) of community-based ART models (i.e. frequency of facility visit, method of ART pick-up, type of health care worker, etc). Additionally, we will select the number of levels (or options) for each attribute (i.e. for frequency of facility visit: every 3 months or every 6 months). These will be used to create a list of all possible combinations. We will divide the subset of combinations chosen in blocks, so that each participant is only asked one possible block of the subset for a total of approximately 10 questions each.

### 3.1.4 Analytic Approach

Qualitative data will be imported into software for managing qualitative analysis (e.g. N-Vivo or Atlas TI) and then subjected to an iterative process of coding using a multi-step process of deductive and inductive techniques. Qualitative data will be cross-referenced to inform and build on the data relating to preferences identified by the quantitative survey and DCSs. Where possible, descriptive statistics and linear regression modeling of scale scores will be utilized to analyze the data. For the discrete choice survey we will estimate main effects plus two-way interactions between different parameters. We will use a nested logit framework for estimation.

### 3.1.5 Sampling Approach/Sample Size Considerations

We have used convenience sampling to determine the number of interviews and focus groups to be conducted (see 1-6 below)

1. Government and community leaders: 20 interviews or until thematic saturation is reached.

2. Professional HCW: two focus group discussions will be conducted (one in an urban clinic and one in a rural clinic) in each province (EP, SP and LP).

3. Lay/Community HCW: two focus group discussions will be conducted (one in an urban clinic and one in a rural clinic) in each province (EP, SP and LP).

4. Patient family members: two focus group discussions will be conducted (one in an urban clinic and one in a rural clinic) in each province (EP, SP and LP).

5. ART patients: four focus group discussions will be conducted (two in an urban clinic and two in a rural clinic) in each province (EP, SP and LP).

6. Pre-Art patients: two focus group discussions will be conducted (one in an urban clinic and one in a rural clinic) in Lusaka province only.

We used the following sample size considerations to determine the number of surveys to be conducted:

General survey: 1,600 patients (of which 800 are ART and 800 are pre-ART).

To derive this sample size we considered a precision approach and calculated the number of ART participants necessary to estimate a binary indicator with 5% margin of error and 95% confidence interval. It is a common practice to assume a prevalence p=0.5 when several indicators are of interest, because p=0.5 is the most conservative choice. We also assume a design effect of 2 since our sampling is clustered at the facility level, and there is significant variability across facilities. Using the formula, we get: n=((1.96^2) * 0.5* 0.5 *2)/(0.05^2)=768, which we approximate at 800. The calculation is repeated for pre-ART patients.

## 3.2 Objective 2: To evaluate the effectiveness, efficiency, and health care quality of a differentiated system of care that includes targeted models of care.

Overall, this objective seeks to evaluate the effectiveness of a differentiated system of HIV care delivery in which the frequency, type, or intensity of contact with the health system is tailored to patient needs. The differentiated system of care includes targeted evaluation of three care models targeting patients stable on ART [community adherence groups (CAGs)], urban adherence groups (UAGs), and fast-track clinic visits), complemented by a model designed for HIV infected patients not yet on therapy (streamlined ART initiation (START)). An overview of these models is available in Appendix 4. These models will be implemented simultaneously, and will be evaluated using a common evaluation framework that includes effectiveness (with a primary outcome of retention), efficiency, and quality.

Patients who meet eligibility criteria, express willingness to participate, and provide written informed consent at the selected intervention sites will be enrolled. Interventions will be targeted by the type of site (rural/urban, high/low volume), baseline retention at site, and by patient status (treatment naïve vs experienced, stability on treatment).

### 3.2.1 Methodology for Objective 2

To evaluate the effectiveness, efficiency and quality of the differentiated care models, a matched cluster randomized design approach is proposed for the CAG and the UAG models, due to substantial weaknesses in existing regional observational pilot data in various settings. Cluster level analysis is being pursued as introduction of the intervention occurs at the clinic level. Randomization is being proposed to account for selection bias encountered in previous observational analyses. As there is little to no observational or implementation data for the Fast-Track and START models, and the goal is to generate such data in Zambia, a difference-in-difference design will be used to calculate the effect of the models on the outcomes of interest.

### 3.2.2 Population

Inclusion criteria:

- HIV-positive adolescents and adults (> 14 years of age)
- Not acutely ill
- For CAGs, UAGs, and Fast-Track models: on ART for at least 6 months
- For the START model: ART naïve and meet the Zambian HIV guidelines for treatment initiation

Exclusion criteria:

- For CAGs, UAGs, and Fast-Track: CD4 < 200 in the last 6 months
- For CAGs, UAGs: Inability to participate in the group activities due to cognition deficits or mental illness.
- Unable to provide consent or unwilling to participate in study
- Pregnancy

###

### 3.2.3 Procedures

1. *Description of Interventions:*

*CAG:* Both rural and urban sites will be eligible for the CAG intervention, consisting of facilitated groups of six people based on geographic proximity of home address and patient preference. This group of six people, will meet monthly at a designated place in the community to provide support and receive medications. Each month one of the members will rotate visiting the clinic for their routine medical visit and will pick up medications for the entire CAG and bring them back to the community. This rotation schedule will recur every six months. The CAG will have a self-selected CAG *leader* within their group to serve as the primary point of contact for the group. They will receive additional support from a facility lay HCW termed a CAG *supervisor.* Patients may be referred to the facility (“up-referred”) for care based on presence of symptoms (acute illness) or patient preference. If up-referred for acute illness, patients will be eligible to continue receiving their ART and adherence support through their CAG while receiving additional clinical care at the facility.

*UAG:* Urban sites will be eligible for the UAG model in which patients will be joined into a UAG group consisting of 30 people. Each UAG group will meet every two to three months at a designated site (either at the clinic facility or another site in the community). Patients will receive (a) group adherence counseling led by a lay HCW (b) two to three month supply of ART medications via a pharmacy tech (c) attendance record and symptom assessment. As in the CAG model, patients may be up-referred for care based on acute illness or patient preference. Patients will continue to visit the facility for a routine medical visit with a professional HCW every six months.

*FAST-TRACK:* Urban sites will also be eligible for the FAST-TRACK intervention. The FAST-TRACK model will provide low-cost additional space (e.g. a mobile unit placed at clinic facility) that will be staffed by a pharmacy technician (to dispense drugs) and two lay health care workers (to provide brief symptom screening to identify patients in need of higher-level care). Patients will be required to visit the FAST-TRACK center every two to three months and will receive ART supply. Data will be captured using the national HIV SmartCare e-card system. Patients will continue to visit the facility for a routine medical visit with a professional HCW every six months.

*START:* Both rural and urban sites will be eligible for the streamlined ART intervention that enables more rapid initiation of ART in order to decrease loss before treatment. Current (2014) Zambian national care and treatment guidelines recommend three structured treatment preparation sessions prior to the initiation of ART for HIV positive non-pregnant adults, adolescents and children. This process can take between 2-4 weeks to complete after determination of ART eligibility. The START model aims to deliver a higher intensity of treatment services by offering same-day CD4 testing and results, streamlined adherence counseling, and quicker initiation of life-long ART to patients enrolling in HIV care and treatment services.

1. *Patient recruitment:*

*At all intervention sites:* The facility staff will identify eligible patients during routine clinic visits and notify the study research assistant about the potential study participant. The research study assistant will assess patient willingness to participate in the intervention and will obtain informed consent for study participation.

For the START model: ART eligibility will be determined by facility staff using national guidelines (excluding pregnant women as they are routinely routed through ANC) and expedited by the provision of on-site CD4 testing using point of care platform after enrollment into the study, for all new HIV infected patients at the facility, and for all returning facility patients that are pre-ART and due for routine CD4 monitoring to determine their eligibility status. Eligible participants will be enrolled into this intervention after they provide informed consent for study participation. No active follow up of participants will be done, after the participant completes the initiation of ART process, during the 12-month follow-up period. At study exit, participants will receive a viral load test as explained below.

*At control sites:* As a cluster randomized study approach is being implemented for CAG and UAG, eligible patients at control sites will be approached for willingness to participate in the intervention. Those who are willing will be enrolled in the study and consent will be obtained to use patient data within SmartCare to evaluate the primary outcome of retention. A viral load measurement will be obtained at baseline and at the end of the follow-up period. No further actions will occur among control site participants and all other study procedures refer only to intervention site participants.

1. *Baseline viral load testing*

For participants enrolled into the CAG and UAG intervention and control groups and into the Fast-Track intervention, dry blood spot specimens will be obtained via a finger prick from each participant for baseline viral load testing after completion of the intake and informed consent process.

At CAG and UAG sites, specimens will be stored and analyzed at the end of the follow-up period. Routine viral load testing, conducted as part of standard clinical care according to national ART treatment guidelines, will be promoted at these sites.

At Fast -Track sites, specimens will be transmitted to the CIDRZ central laboratory for testing and results will be communicated to clinics (using current standard procedures for laboratory results reporting) and to study staff.

Patients who are found to not be virologically suppressed (defined as viral load > 1,000 copies/mL) will remain eligible to continue to participate in the Fast-Track model though they will be scheduled for additional visits to the clinic based on national ART treatment guidelines as well as receive intensified support from the lay HCW.

1. *Baseline CD4 testing*

For intervention participants enrolled into the CAG, UAG and Fast-Track models, study staff will liaise with the clinic to facilitate baseline CD4 testing after completion of the intake and informed consent process.

Specimens will be transmitted to the CIDRZ central laboratory for testing and results will be communicated directly to clinics (using current standard procedures for laboratory results reporting) and to study staff. Patients with CD4 count <200 will remain eligible to continue to participate in the models but may receive additional visits to the clinic at the discretion of the provider according to current standard of care.

1. *Costing*

Information will be collected during the intervention period on a subset of participants in each model using a semi-structured questionnaire to assess direct and indirect health costs of the intervention. Administrative data, where needed, will also be used.

1. *Exit viral load testing and patient exit-survey*

In all four models, patients will have an exit viral load test performed at the end of the 12-month intervention period. In CAG, UAG, and Fast-Track models, participants will be administered a patient exit-survey to assess patient satisfaction and patient centeredness.

A summary of the routine schedule of activities within four models of differentiated care is shown in Appendix 5.

### 3.2.4 Measurements

We will use information from routine clinical documentation, which includes:

1. Socio-demographic, laboratory, clinical, and pharmacy data

We will extract standard de-identified socio-demographic, laboratory and clinical data for all patients (intervention and control) from the patient’s electronic medical records.

1. Participant attendance, individual ART pick-up, and symptom assessment for UAG, CAG, and Fast-Track

We will use model specific registers that include information such as 1) Attendance, 2) Receipt of medications 3) Symptom checklist 4) Whether patient prefers to remain in care model or return to standard clinical care. This information will be entered into the study database using a tablet.

In addition, the study will also measure additional processes and outcomes related to each of the four models of care, which includes:

1. Movement between tiers for UAG, CAG and Fast-Track

The presence of symptoms or a patient’s desire to return to standard care will trigger an up-referral to the facility. Patients with acute illness, once stabilized, will be down-referred back to the model. Movement between tiers will be measured using a register that will document any up-referral after initial study enrollment. This information will be entered into the study database using a tablet. Down-referrals will be captured by measuring assigned frequency of clinic visit in the electronic medical record.

1. Patient satisfaction and patient-centeredness for UAG, CAG and Fast-Track

Patient satisfaction and centeredness will be measured as part of a semi-structured patient-exit survey that will be conducted after the 12-month intervention period.

1. Viral load testing

Viral load testing (at baseline and exit) using dried blood spot specimens will be conducted as noted in the procedures section.

1. Costing

A semi-structured costing questionnaire will be administered to a subset of intervention participants to assess direct and indirect health costs of the intervention (as noted in the procedures section). In addition, to calculate the resource use of each activity, we will use time-in-motion studies among patients and health care workers prior to and during the intervention.

1. Process data will be collected through qualitative assessments (i.e. HCW interviews during and at the end of follow-up period) and facility-level assessments (i.e. frequency of pharmacy stock outs).

### 3.2.5 Analytic Approach and Sample Size Considerations

*a. Outcomes and Analysis*

The primary outcome in three models that target retention in care (CAG, UAG, Fast-track) is defined as time to first missed pharmacy pick-up (> 7 days late). For the START model which targets ART initiation, we use the date of ART initiation as the primary outcome. To evaluate the impact of CAG and UAG interventions, we will use Kaplan-Meier estimates of time to the primary outcome, stratified by intervention condition, using log rank tests. For Fast-track and START, our primary analysis will take a difference-in-difference approach in which we estimate the difference in the change in the rate of the outcome between intervention and control settings. In secondary analyses of the primary outcome, we will explore longer intervals of lateness that comprise a missed pharmacy visit (14 and 30 days) as well as adjustment for baseline patient characteristics. We will also explore analysis using medication possession ratio (MPR) as the outcome and other metrics of retention ^17^ that incorporate both frequency of missed visit as well as interval of time before return.

The secondary outcomes will focus on implementation and quality (see Appendix 2).

a. *Implementation Outcomes*

- *Feasibility* will be evaluated qualitatively through analysis of interviews and focus groups.
- *Costs* will be evaluated quantitatively using “bottom-up” activity-based micro-costing techniques (see Measurements section above) and “top-down” evaluation of budgets and spending reports, based on calculations of DALY’s.
- *Fidelity* of the intervention models will be assessed through estimates of the proportion of identified and eligible patients successfully enrolled in a model, the proportion of missed visits, and the proportion of CAG/UAG still functioning at the end of the 12-month intervention period.

*b. Quality Outcomes*

We use the framework offered by the Institute of Medicine to define quality outcomes.:

- *Access* will be evaluated using time (days) from positive symptom screen to appropriate referral.
- *Efficiency* will be assessed using cost-effectiveness analysis. We will consider the incremental cost per additional patient retained and the cost per death averted using effectiveness data from each model.
- *Safety* will be evaluated by estimates of the proportion of patients with appropriate laboratory monitoring and symptom screening.
- *Equity* will be assessed by comparing retention rates between adults and adolescents and men and women.
- *Patient-centeredness* will be assessed through comparison of means in the results of appropriate dimensions in the semi-structured patient exit-survey.

*c. Patient Outcomes*

- HIV RNA: we compare the proportion of patients suppressed at one year among those exposed to the intervention and control conditions using mixed effects logistic regression
- Satisfaction: we will compare the effect of the intervention on patient satisfaction through mixed effects linear regression.

1. *Sample Size Considerations*

- CAG/UAG: Existing program data suggest that 65% of patients are at least 7 days late for a pharmacy refill visit during their first year in HIV care after starting ART and that 95% of clinics fall within 15% (that is 50 to 80) of this estimate. Thus, a conservative matched co-efficient of variation is estimated to be 0.10. We assume a 50% reduction in missed pharmacy visits due to CAG and UAG. Under these assumptions, for the CAG intervention, our selection of five clinics and 120 patients per site (in 20 CAGs per site assuming complete enrollment), the study will be powered at 96%. Under more conservative assumptions about between clinic variability and the effect size (Appendix 6) this sample size yields power of over 80%. For the UAG intervention, our target sample size is 4 UAGs at each of the five intervention sites with 30 people in each UAG and therefore 120 participants per site (total of 600 participants across sites). Under the same assumptions about coefficient of variation and the effect size as for the CAG model, this sample size yields power of > 90% and is robust to varying assumptions about correlation (See Appendix 6).
- Fast-track: Our target sample size for Fast-track is 200 patients at each of four sites (two intervention sites and two control sites; n=800 total). Assuming baseline 80% of patients miss a pharmacy visit in a year of care, which we anticipate will fall to 40%, we have 80% power.
- START: Assuming ART initiation at 2 weeks rises from 60% to 85%, 100 patients at each of eight sites (four intervention and four control sites; n=800 total), rho of 0.15 yields an anticipated power of 78%. No more than 200 patients will be enrolled into the study to receive POC CD4 testing with their consent. Enrolment will stop when we reach 100 patients who are eligible for ART initiation.

No control patients will be actively enrolled in START and Fast Track sites. We will use routinely collected, de-identified, aggregated data to evaluate the primary outcome of retention (Fast Track) and days to ART initiation (START).

### 3.2.6 Study Timeline

For all interventions with the exception of the START model, enrollment will occur for a four-six months period (Months 10-15 on Study Timeline). Enrollment will begin on Day 1 of the enrollment period and will stop either after the target number of patients are enrolled or at the end of the four-six months enrollment period (whichever comes sooner). Implementation will occur for a twelve-month period (Months 16-27 on Study Timeline). Outcome (retention) data will be measured at Month 27 of the study.

## 3.3 Objective 3: Methodological Toolkit

After analysis and synthesis of data collected during Objectives 1 and 2, the study team will develop a methodological toolkit that can be used to assess the feasibility, acceptability, needs, and preferences for differentiated models of care in other contexts and provide guidance for implementation of each model. Data will be collected using work process tracking and review with study staff members; cost tracking; and interviews with study staff and key decision makers. We will describe the processes required for assessing community ART needs, including identification of appropriate stakeholders for IDIs and FGDs, analysis of time taken to complete all data collection with a focus on understanding the key resources, and other inputs necessary to replicate these activities. Work process review will include quantification/counting of work activities and timing. Cost data will be tracked according to required accounting procedures. Qualitative data, including interview transcripts capturing information on the interactions between HIV patients and professional and lay health care workers that support various models of care, will be analyzed using grounded theory methods including the application of inductive and deductive codes. In addition, the toolkit will be informed by structured consultations (e.g. debriefing sessions) with qualitative research assistants and survey/DCE enumerators to identify key challenges encountered in the data collection process. The toolkit will be designed to be easily digestible and result in actionable plans to move from current standards to viable differentiated care models in a variety of circumstances and contexts. Based on input from reviewers, we will finalize the toolkit and provide it in both a downloadable digital and hard copy form with dissemination events with the MoH, MCDMCH and other appropriate national and sub-national stakeholders.

# 6.0 Reference List

1. Health ZMo. *UNAIDS Country Progress Report Zambia.*  March 31, 2012 2012.

2. Humphreys CP, Wright J, Walley J, et al. Nurse led, primary care based antiretroviral treatment versus hospital care: a controlled prospective study in Swaziland. *BMC health services research.* 2010;10:229.

3. Jaffar S, Amuron B, Foster S, et al. Rates of virological failure in patients treated in a home-based versus a facility-based HIV-care model in Jinja, southeast Uganda: a cluster-randomised equivalence trial. *Lancet.* 2009;374(9707):2080-2089.

4. Kipp W, Konde-Lule J, Saunders LD, et al. Antiretroviral treatment for HIV in rural Uganda: two-year treatment outcomes of a prospective health centre/community-based and hospital-based cohort. *PloS one.* 2012;7(7):e40902.

5. Kredo T, Ford N, Adeniyi FB, Garner P. Decentralising HIV treatment in lower- and middle-income countries. *The Cochrane database of systematic reviews.* 2013;6:CD009987.

6. Luque-Fernandez MA, Van Cutsem G, Goemaere E, et al. Effectiveness of patient adherence groups as a model of care for stable patients on antiretroviral therapy in Khayelitsha, Cape Town, South Africa. *PloS one.* 2013;8(2):e56088.

7. Decroo T, Koole O, Remartinez D, et al. Four-year retention and risk factors for attrition among members of community ART groups in Tete, Mozambique. *Tropical medicine & international health : TM & IH.* 2014;19(5):514-521.

8. Decroo T, Telfer B, Biot M, et al. Distribution of antiretroviral treatment through self-forming groups of patients in Tete Province, Mozambique. *Journal of acquired immune deficiency syndromes.* 2011;56(2):e39-44.

9. Fairall L, Bachmann MO, Lombard C, et al. Task shifting of antiretroviral treatment from doctors to primary-care nurses in South Africa (STRETCH): a pragmatic, parallel, cluster-randomised trial. *Lancet.* 2012;380(9845):889-898.

10. Brennan AT, Long L, Maskew M, et al. Outcomes of stable HIV-positive patients down-referred from a doctor-managed antiretroviral therapy clinic to a nurse-managed primary health clinic for monitoring and treatment. *Aids.* 2011;25(16):2027-2036.

11. Babigumira JB, Castelnuovo B, Stergachis A, et al. Cost effectiveness of a pharmacy-only refill program in a large urban HIV/AIDS clinic in Uganda. *PloS one.* 2011;6(3):e18193.

12. Selke HM, Kimaiyo S, Sidle JE, et al. Task-shifting of antiretroviral delivery from health care workers to persons living with HIV/AIDS: clinical outcomes of a community-based program in Kenya. *Journal of acquired immune deficiency syndromes.* 2010;55(4):483-490.

13. Bemelmans M, Baert S, Goemaere E, et al. Community-supported models of care for people on HIV treatment in sub-Saharan Africa. *Tropical medicine & international health : TM & IH.* 2014;19(8):968-977.

14. Rosen S, Fox MP. Retention in HIV care between testing and treatment in sub-Saharan Africa: a systematic review. *PLoS Med.* 2011;8(7):e1001056.

15. Frontieres MS. Community ART Group Toolkit: How to Implement the CAG Model. <http://www.msf.org/sites/msf.org/files/cag_toolkit.pdf>. Accessed August 28, 2014.

16. Rasschaert F, Telfer B, Lessitala F, et al. A qualitative assessment of a community antiretroviral therapy group model in Tete, Mozambique. *PloS one.* 2014;9(3):e91544.

17. Hickey MD, Salmen CR, Omollo D, et al. Implementation and Operational Research: Pulling the Network Together: Quasiexperimental Trial of a Patient-Defined Support Network Intervention for Promoting Engagement in HIV Care and Medication Adherence on Mfangano Island, Kenya. *Journal of acquired immune deficiency syndromes.* 2015;69(4):e127-134.

# 7.0 Appendices

## 7.1 Appendix 1: Selected Differentiated Care Model Outcome Studies

| **Study & Year (Please refer to References above for details)** | **Country** | **Study type** | **General strategy** | **Differentiated Model Element** |
| --- | --- | --- | --- | --- |
| Jaffar 2009 | Uganda | Cluster randomized trial | Down-referral to home (decentralization) and care by trained field officer (task shifting) every month compared to HIV clinic visit by MD every two months  Up-referral for positive symptom or adverse effect screen. | Service location  Health-worker cadre  Service frequency |
| Humphreys 2010 | Swaziland | Non-randomized trial | Down-referral to primary health center (decentralization) and care by nurse (task shifting) compared to hospital based care by MD  Up-referral when RN had questions about care | Service location  Health-worker cadre |
| Selke 2010 | Kenya | Cluster randomized trial | Down-referral to home (decentralization) and care by peer with PDA support (task shifting) every three months compared to HIV clinic visit with RN every month  Up-referral if PDA triggered alarm based on inputted symptoms, vital signs, pill count | Service location  Health-worker cadre  Service frequency |
| Babigumira 2011 | Uganda | Retrospective cohort | Down-referral to receive ART by pharmacy based nurse (task shifting) every month  Up-referral for “major clinical or social problems” or non-adherence | Health-worker cadre |
| Brennan 2011 | South Africa | Propensity matched retrospective cohort | Down-referral to primary health center (decentralization) and care by nurse (task shifting) every 2 months vs HIV clinic visit with MD every 6 months.  Up-referral for missed clinic visit or detectable viral load | Service location  Health-worker cadre  Service frequency |
| Fairall 2012 | South Africa | Cluster randomized trial | Down-referral to primary health center (decentralization) and care by nurse (task shifting) compared to hospital HIV clinic by MD  Up-referral based on criteria specified in national guidelines | Service location  Health-worker cadre |
| Kipp 2012 | Uganda | Prospective cohort | Down-referral to home (decentralization) and care by community volunteers (task shifting) every week.  Up-referral for positive symptom or adverse effect screen. | Service location  Health-worker cadre  Service frequency |
| Luque-Fernandez 2013 | South Africa | Retrospective cohort | Down-referral to community (decentralization) and care by peer or lay counsellor (task shifting) every 2 months | Service location  Health-worker cadre |

## 7.2 Appendix 2: Outcomes Framework

##
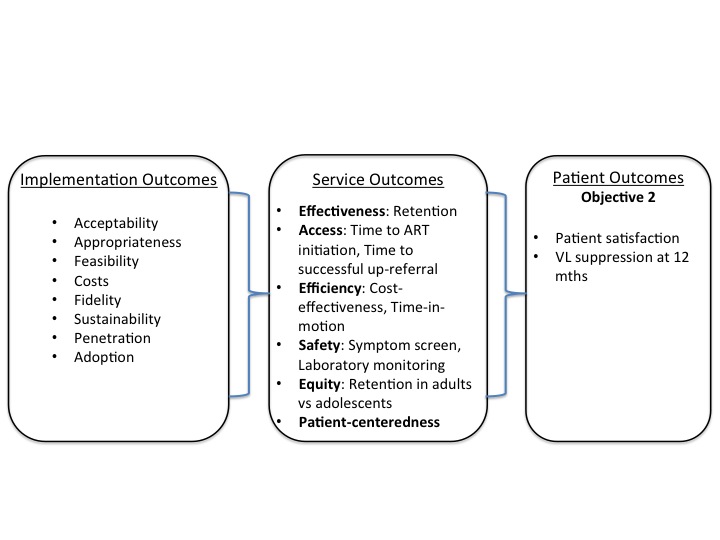


## 7.3 Appendix 3: Objective 1 Overview

| **Study Population** | **Measurements** | **Number of Sites/Sampling Approach** | **Sample Size** |
| --- | --- | --- | --- |
| Government and community leaders | In-depth interviews (qualitative) | - National: Individuals from MOH, MCDMCH, CMS, Central Laboratory - District Health Officers - Local community leaders - Implementing partners/civil society organizations | 20 interviews or until thematic saturation reached |
| Professional health care workers (doctor, nurse, pharmacist, clinic in-charge) | Focus Group Discussion (qualitative) | - Within each of three provinces (Lusaka, Southern, Eastern): 2 FGDs (1 Urban & 1 Rural) - 1 In-charges only FGD in Lusaka Urban District | 8 participants per FGD x 7 FGDs= 56 total participants |
| Lay/community health care workers | Focus Group Discussion (qualitative) | - Within each of three provinces (Lusaka, Southern, Eastern): 2 FGDs (1 Urban & 1 Rural) | 8 participants per FGD x 6 FGDs= 48 total participants |
| Family members of PLWHIV | Focus Group Discussion (qualitative) | - Within each of three provinces (Lusaka, Southern, Eastern): 2 FGDs (1 Urban & 1 Rural) |  |
| ART patients | Focus Group Discussion (qualitative) | - Within each of three provinces (Lusaka, Southern, Eastern): 4 FGDs (2 Urban & 2 Rural) | 8 participants per FGD x 12 FGDs= 96 total participants |
|  | General survey (quantitative) | - Within each of three provinces (Lusaka, Southern, Eastern): 2 urban & 2 rural clinics - Random sample of patients visiting a survey clinic will be approached for participation. | 800 |
|  | Medical record review (quantitative) | Same as for general survey | Same as for general survey |
|  | Discrete choice survey (quantitative) | - The first 500 patients consented to complete the general survey will be asked to complete the discrete choice survey. | 500 |
| Pre-ART patients | Focus Group Discussion (qualitative) | - Within Lusaka province only: 2 FGDs (1 Urban & 1 Rural) | 8 participants per FGD x 2 FGDs= 16 total participants |
|  | General survey (quantitative) | - Within each of three provinces (Lusaka, Southern, Eastern): sampling will occur at 2 urban and 2 rural clinics - Random Sample of patients visiting a survey clinic will be approached for participation. | 800 |
|  | Medical record review (quantitative) | Same as for general survey | Same as for general survey |

## 7. 4 Appendix 4: Overview of four models of differentiated care implemented in Objective 2

| **Model** | **Location** | **Number of sites** | **Number groups per site** | **Total sample size** | **Problem(s) addressing** | **Description of model** | **Selected related literature** |
| --- | --- | --- | --- | --- | --- | --- | --- |
| Model 1: Community adherence groups (CAGs) | Rural | 5 | 20 groups of 6 patients | 600 | Poor adherence and retention; long patient travel time and cost; rural clinic congestion and wait time | Monthly patient ART groups, facilitated by lay counselor, meet at location of group’s choice. Patients travel to clinic once every six months (rotating between group members) for labs and to pick up medications. | Decroo et al, 2011  Rasschaert et al, 2014 |
| Model 2: Urban adherence groups (UAGs) | Urban | 5 | 4 groups of 30 patients | 600 | Poor adherence and retention; long patient wait time and cost; urban clinic congestion | 2-3 monthly patient ART groups, meet during off-hours at clinics. Adherence discussion by lay ART counselor; ARVs distributed by pharmacy technician. Patients schedule clinic visit once every six months. | Luque –Fernandez, 2013 |
| Model 3: FAST-TRACK | Urban | 2 | N/A | 400 | Urban clinic space limitations/ congestion; wait time; poor retention | Container unit or dedicated room at clinics provide ART patients with rapid refills and health checks, facilitated by use of electronic health card. | McGuire et al, 2011  CIDRZ/MIT operational observation |
| Model 4: START | Urban & rural | 4 | N/A | 400 | Poor retention in newly eligible patients; clinic congestion and wait time | Patients testing positive will be given an option to move into a streamlined process wherein substantial counseling is delivered, as well as necessary baseline safety laboratory tests and ART is provided as soon as possible after being classified as eligible to initiate ART per guidelines. | Karim, 2009  Blanc, 2010  Amuron, 2009  Geng, 2011 |

## 7.5 Appendix 5: Routine schedule of activities within four models of differentiated care implemented in Objective 2

| Model | Frequency of Activity (interaction) | Symptom Screen | Interaction with clinician | Drug dispensation | Primary HCW Interaction |
| --- | --- | --- | --- | --- | --- |
| 1: Community Adherence Group | Monthly | Every visit | Every 6 months or acute event | Monthly | Lay health care worker |
| 2: Urban adherence Group | Every 2-3 months | Every visit | Every 6 months or acute event | Every 2-3 months | Lay health care worker |
| 3: Fast-Track | Every 3 months | Every visit | Every 6 months or acute event | Every 3 months | Pharmacy technician |
| 4: START | Intense initial ART facility interaction until ART initiation | Every visit | Every visit until ART initiation | At initiation | Clinical officer |

## 7.6 Appendix 6: Power calculations for Objective 2: CAG and UAG

| Alpha | Pi0 | Pi1 | K | J | N | Power |
| --- | --- | --- | --- | --- | --- | --- |
| **Modify Pi0 and Pi1** |  |  |  |  |  |  |
| 0.05 | **0.8** | **0.6** | 0.1 | 30 | 5 | 0.62 |
| 0.05 | **0.8** | **0.4** | 0.1 | 30 | 5 | 0.997 |
| 0.05 | **0.7** | **0.5** | 0.1 | 30 | 5 | 0.63 |
| 0.05 | **0.6** | **0.4** | 0.1 | 30 | 5 | 0.66 |
| 0.05 | **0.6** | **0.3** | 0.1 | 30 | 5 | 0.96 |
| 0.05 | **0.4** | **0.2** | 0.1 | 30 | 5 | 0.7989 |
|  |  |  |  |  |  |  |
| **Modify k** |  |  |  |  |  |  |
| 0.05 | 0.6 | 0.3 | **0.1** | 30 | 5 | 0.96 |
| 0.05 | 0.6 | 0.3 | **0.2** | 30 | 5 | 0.82 |
| 0.05 | 0.6 | 0.3 | **0.3** | 30 | 5 | 0.597 |
|  |  |  |  |  |  |  |
| **Modify j** |  |  |  |  |  |  |
| 0.05 | 0.6 | 0.3 | 0.1 | **10** | 5 | 0.64 |
| 0.05 | 0.6 | 0.3 | 0.1 | **15** | 5 | 0.7988 |
| 0.05 | 0.6 | 0.3 | 0.1 | **30** | 5 | 0.96 |
| 0.05 | 0.6 | 0.3 | 0.1 | **45** | 5 | 0.991 |
| 0.05 | 0.6 | 0.3 | 0.1 | **90** | 5 | 0.999 |
| 0.05 | 0.6 | 0.3 | 0.1 | **120** | 5 | 0.999 |
|  |  |  |  |  |  |  |
| **Modify n** |  |  |  |  |  |  |
| 0.05 | 0.6 | 0.3 | 0.1 | 30 | **5** | 0.96 |
| 0.05 | 0.6 | 0.3 | 0.1 | 30 | **6** | 0.99 |
| 0.05 | 0.6 | 0.3 | 0.1 | 30 | **7** | 0.997 |

alpha= significance level

Pi0= baseline proportion

Pi1=post-intervention proportion

k=co-efficient of variation

j= sample size per cluster

n= number of matched cluster
